# Maize leaf disease identification based on WG-MARNet

**DOI:** 10.1371/journal.pone.0267650

**Published:** 2022-04-28

**Authors:** Zongchen Li, Guoxiong Zhou, Yaowen Hu, Aibin Chen, Chao Lu, Mingfang He, Yahui Hu, Yanfeng Wang

**Affiliations:** 1 College of Computer & Information Engineering, Central South University of Forestry and Technology, Changsha, Hunan, China; 2 Plant Protection Research Institute, Academy of Agricultural Sciences, Changsha, Hunan, China; 3 National University of Defense Technology, Changsha, Hunan, China; Politechnika Slaska, POLAND

## Abstract

In deep learning-based maize leaf disease detection, a maize disease identification method called Network based on wavelet threshold-guided bilateral filtering, multi-channel ResNet, and attenuation factor (WG-MARNet) is proposed. This method can solve the problems of noise, background interference, and low detection accuracy of maize leaf disease images. To begin, a processing layer called Wavelet threshold guided bilateral filtering (WT-GBF) based on the WG-MARNet model is employed to reduce image noise and perform high and low-frequency decomposition of the input image using WT-GBF. This increases the input image’s resistance to environmental interference and feature extraction capability. Secondly, for the multiscale feature fusion technique, an average down-sampling and tiling method is employed to increase feature representation and limit the risk of overfitting. Then, on high and low-frequency multi-channel, an attenuation factor is introduced to optimize the performance instability during training of the deep network. Finally, when the convergence and accuracy are compared, PRelu and Adabound are used instead of the Relu activation function and the Adam optimizer. The experimental results revealed that our method’s average recognition accuracy was 97.96%, and the detection time for a single image was 0.278 seconds. The average detection accuracy has been increased. The method lays the groundwork for the precise control of maize diseases in the field.

## 1. Introduction

Maize is an important food crop and one of the most widely planted food crops globally. A healthy maize industry plays an essential role in ensuring the world’s food security. However, with the change in climate and environment, the stress of diseases and insect pests caused irreversible losses to maize and other crops. The leaf diseases caused by various pathogens greatly restricted the photosynthesis of maize leaves and the transportation of nutrients, which seriously affected the yield and quality of maize [1]. Only symptomatic spraying of pesticides can control the spread of diseases and minimize losses. It is essential for disease control to discover and judge the disease type in the time and select suitable pesticides for precise treatment. The traditional method of relying on plant pathologists to identify disease types on-site is time-consuming, labor-intensive, inefficient, and prone to subjective errors, especially in the field environment, which greatly increases labor costs [2]. In recent years, machine vision combined with image processing technology is continuously overcoming the shortcomings of artificial recognition, such as easy misjudgment, dependence on expert experience, labor, and manpower [3, 4]. However, the ideas of these studies are often based on the color, texture and spatial structure of the image [5, 6]. The thresholds cannot meet all complex background images obtained under natural conditions, and the ultimate recognition accuracy is limited. There are some problems such as poor adaptability, weak anti-jamming ability, etc. As a result, the practical application is severely limited.

At present, more and more researchers are devoted to the field of deep learning. Compared with the traditional recognition methods, the emergence of the convolutional neural network (CNN) effectively improves the recognition efficiency and accuracy, which is obviously better than machine vision. Since the proposal of LeNet [7] in 1998, the convolutional neural network has developed continuously upgraded models such as AlexNet [8] in 2012, Googlenet [9] in 2014, and ResNet [10] in 2015. Many novel CNN models are also being proposed to apply in the field of plant classification. For example, Muhammad Rizwan Latif [11] proposed using deep learning architecture, serial feature fusion, and the optimal feature selection convolutional neural network. Nazar Hussain et al. [12] proposed a new deep learning-based framework for plant leaf disease identification that includes feature fusion and selecting the best features.

The network models become more and more deep and complex. At the same time, it also solves the problems of gradient disappearance and the explosion of backpropagation. However, in practice, the original disease images of maize leaves include issues such as noise and background interference, resulting in low classification accuracy. In order to address the aforementioned issues, we propose a WG-MARNet-based maize disease identification method capable of identifying maize leaf pests and diseases.

The following are our two main contributions.

1. We use data augmentation to improve data quality, diversify data features, and expand the size of our dataset to achieve a better outcome.
2. We propose the WG-MARNet with the following design for the classification of maize leaf diseases.
  - To minimize image noise at the input side and to construct a high and low frequency multi-channel network structure, a wavelet threshold-guided bilateral filtering (WT-GBF) algorithm is proposed to be integrated into the network structure based on the features of maize diseases.
  - It is proposed to use average down-sampling and tile operations to improve the multi-scale feature fusion technology. Use improved multi-scale feature fusion technology to enhance the ability of target feature expression.
  - An attenuation factor is proposed to be added to the high and low-frequency channels to increase the stability of the network parameters during learning.

To make it easier for the reader to read this paper, Table 1 lists the abbreviations that frequently appear in this paper.

**Table 1:** Abbreviations appearing in this article.

Abbreviations	Full Name
WG-MARNet	Network based on wavelet threshold-guided bilateral filtering, multi-channel ResNet, and attenuation factor
WT-GBF	Wavelet threshold guided bilateral filtering
LDP	local discriminant projects
ISDA	Integrated Subspace Discriminant Analysis
SSD	Single Shot MultiBox Detector
SGD	Stochastic Gradient Descent
DHLD	Denoising and High and Low-frequency Decomposition module
MFF	Multi-scale Feature Fusion module
MCFF	Multi-channel Feature Fusion module
AF	Addition of the attenuation Factor module
IoT	Internet of Things

## 2. Related work

In recent years, with the application of computer vision technology in the field of agriculture, there have been a lot of achievements in the research of image segmentation based on crop and disease location. In 2010, Hengqiang Su et al. [13] extracted the lesion area of maize leaf by Super-Green segmentation and Otsu threshold segmentation, combined with image morphology operation, then extracted the color and texture characteristics parameters of different lesion areas of maize leaf, and finally classified the experimental data by SVM method. In 2012, in order to better identify common types of maize diseases, Baiyi Zhang [14] partitioned the lesion area by preprocessing and using an improved level set algorithm and achieved a good recognition accuracy. In 2013, in order to identify maize varieties, Donglaima et al. [15] first used the Ostu algorithm to segment maize varieties, on which six characteristic parameters were extracted, and then used the K-means clustering algorithm to identify maize varieties. In 2014, in order to better identify maize diseases, Shanwen Zhang et al. [16] proposed an algorithm of local discriminant projects (LDP) to identify maize diseases. Based on image segmentation and LDP, the dimension of the disease image is reduced. Finally, a database is established to identify the disease image with high accuracy. In 2016, in order to better identify maize leaf diseases, Liangfeng Xu et al. [17] proposed an adaptive multi-classifier method to identify maize leaf diseases, and combined it with cluster analysis to obtain adaptive weights. The proposed method can improve the accuracy of maize leaf disease identification. However, because of the weak robustness of traditional support vector machine and other methods, the application effect is not good in a complex field environment. Also, some researchers have contributed in terms of data. To help farmers with the early detection of plant diseases and other scholars with data from their research, Hafiz Tayyab Rauf et al. [18] made a dataset containing diseases of citrus fruits such as Healthy, Blackspot, Canker, Scab, Greening, and Melanose.

With the deep learning technology in target detection and image processing, convolutional neural network (CNN) has been widely used in image recognition and classification [19, 20]. In the research of plant diseases and insect pests identification, CNN has been proved to have better performance than traditional machine learning methods. Brahimi et al. [19] used 15,000 tomato disease images to classify and recognize 9 diseases in the data set based on the AlexNet model and obtained better recognition results. In 2017, Mansheng Long et al. [21] applied migration learning in the convolution network training process, constructed AlexNet model based on TensorFlow, and classified algae spot, yellow disease, coal pollution disease, and soft rot disease of Camellia oleifera with 96.53% accuracy. Rehman M Z U [22] proposed a new technique in 2018 for apple and grape disease detection and classification based on new adaptive thresholding and optimized weighted based segmentation fusion. The method is highly efficient in terms of accuracy, sensitivity, precision, and F1 value. Muhammad Zia Ur Rehman [23] proposed a classification method in 2021 for citrus diseases based on deep learning which achieved 95.7% classification accuracy. In 2021, Jaweria Kianat [24] proposed a framework for cucumber disease classification based on feature fusion and selection techniques based on deep learning with an accuracy of 93.50% obtained from the selected dataset. Ahmad Almadhor et al. [25] trained advanced classifiers for image-level and disease-level classification using a high-resolution guava leaf and fruit dataset and obtained an overall classification accuracy of 99%. Almetwally M. Mostafa et al. [26] proposed an AI-Driven framework for the recognition of guava plant diseases through machine learning. After pre-processing and enhancing the data, enhanced data were then augmented over the nine angles using the affine transformation method—augmented enhanced data used by five DL networks by altering their last layers. The final application was applied to different networks and better results were obtained. In 2022, Zia ur Rehman [27] proposed a new method for real-time apple leaf disease detection and classification using MASK-RCNN and deep learning feature selection based on deep learning, achieving the best accuracy of 96.6% in Integrated Subspace Discriminant Analysis (ISDA) classification.

Although the above research has produced positive results, most of the previous researches on methods of identifying disease types with the help of deep learning and CNN were carried out in the laboratory or under controlled conditions. The sample size of the image set obtained in the field is small, which affects the generalization of the model. When using a large public data set as a research object, the image background in the data set is too simple and the data is seriously underrepresented. In the face of practical application, due to the lack of representativeness of the data set, the ability of the model to extract disease regional features in the complex background is reduced, especially in the actual maize original image, such as noise, unclear features, and background interference, the recognition accuracy, and speed are greatly reduced.

Facing the above problems, we proposed WG-MARNet for the classification of maize leaf disease. The method can eliminate the noise of maize images, enhance the focus characteristics of maize, and realize the high-precision recognition of maize disease images.

**Fig 1:**
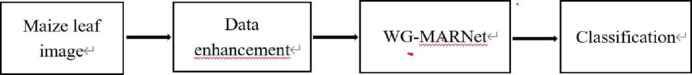
Flowchart of data enhancement and WG-MARNet framework.

1. The improvement of the WG-MARNet is as follows:
  - According to the principle that maize lesions have huge feature differences in high and low frequency images, the wavelet threshold-guided bilateral filtering is used for high and low frequency decomposition, and a high and low frequency multi-channel network structure is established to improve the ability of feature extraction.
  - The multi-scale feature fusion method is improved by using average down-sampling and tile operations. This not only enhances the ability of target feature expression but also reduces the increase in the number of features and reduces the risk of overfitting.
  - Attenuation factors are introduced on the high and low frequency multi-channels to optimize the problem of unstable performance when training deep networks.
  - Through the comparative experiments of convergence and accuracy, we use PRelu and Adabound instead of the Relu activation function and Adam optimizer.
2. The flow chart based on data enhancement and the WG-MARNet framework is shown in Fig 1.

## 3. Materials and methods

### 3.1 Data acquisition and preprocessing

The data set used in the experiment was derived from the data set website and field collection. Websites for collecting the data set include the China Science Data Network (http://www.csdata.org/) and Digipathos. On the data set website, 150 images of 9 common maize diseases caused by fungi are carefully selected. The other part of our data set is collected in cooperation with the Hunan Academy of Agricultural Sciences, China. We use Sony ILCE-7M2 to shoot optical images of different diseases from multiple angles at different time periods in the morning, noon, and evening under sunny and cloudy weather conditions. Such photos can reflect the many complex conditions of maize growing in the field to ensure that the collected images are more representative. Finally, 1000 images were collected with a pixel size of 3600×2700, including 458 samples with uniform illumination under sunny conditions, 263 samples with uneven illumination, and 279 samples under cloudy conditions. The number of 9 disease images finally obtained through field collection and data collection website is 1150. https://github.com/FXD96/Corn-Diseases is the link to the dataset we collated.

In order to effectively improve data quality, increase the diversity of data features, and reduce the dependence of convolutional networks on computer hardware due to complex backgrounds, data enhancement operations are performed on the collected disease image sets. we use multi-angle flipping, brightness adjustment, saturation adjustment, and adding Gaussian noise to expand the corresponding data set to 8 times the original. The transformed image is uniformly adjusted to 224×224×3 (height×width×color channel). The original sample size and the enhanced sample size distribution are shown in Table 2.

**Table 2:** Profile of sample images for nine types of diseases.

Category	Number of Original samples	Number of Augmented samples
Anthracnose leaf blight	107	856
Tropical rust	115	920
Southern maize rust	130	1040
Common rust	142	1136
Southern leaf blight	150	1200
Phaeosphaeria leaf spot	120	960
Diplodia leaf streak	116	928
Physoderma brown spot	128	1024
Northern leaf blight	142	1136

### 3.2 WG-MARNet

In order to improve the recognition accuracy of maize diseases and solve the problem of low accuracy caused by noise and unclear features in original maize images obtained from the complex environment, this paper designs a WG-MARNet model.

**Fig 2:**
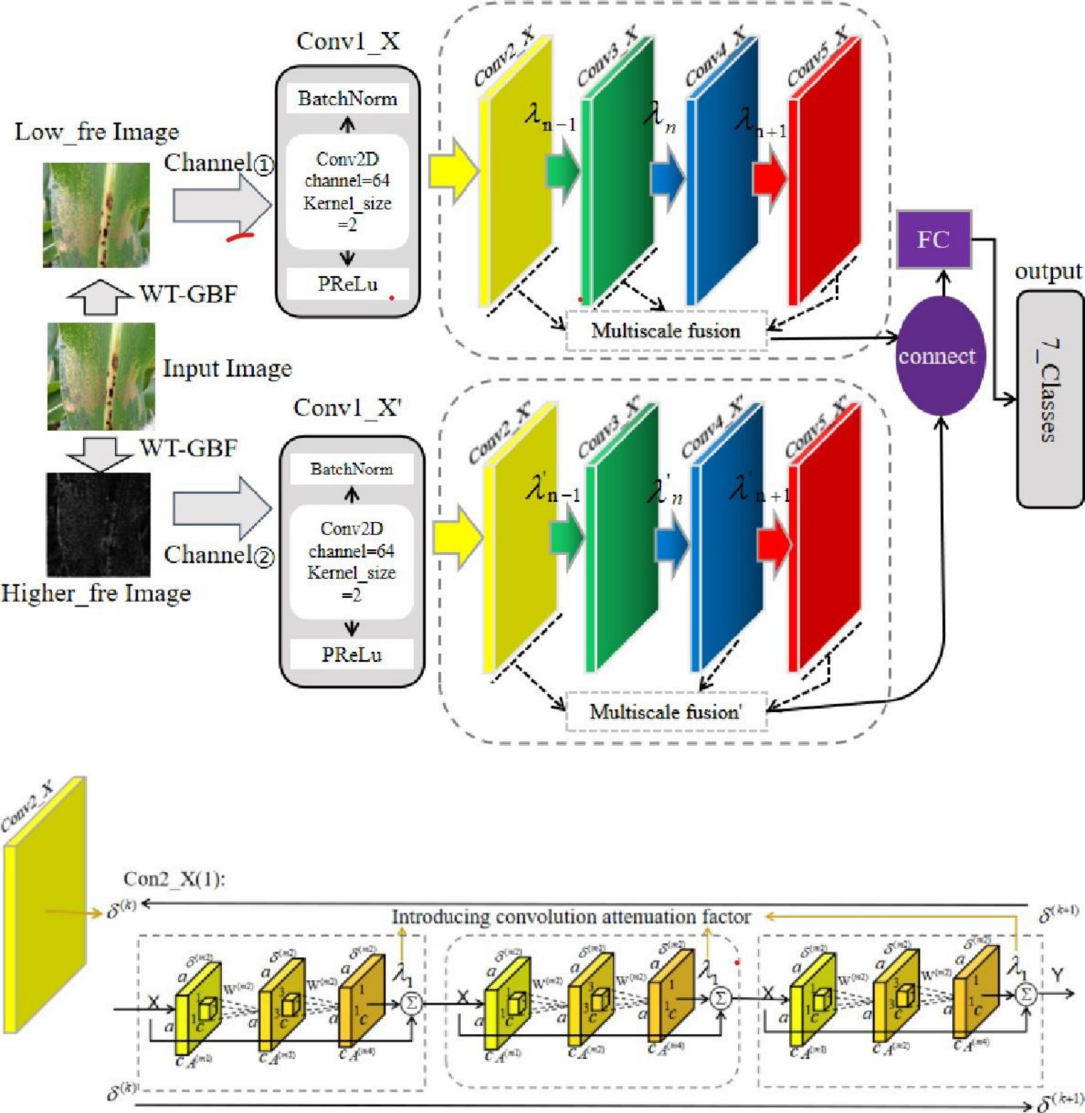
Structure diagram of the WG-MARNet.

First, the maize leaf disease image data set is used as the input of the model. After the WT-GBF processing layer, the image noise is eliminated and the input image is decomposed at high and low frequencies, which improves the ability to resist environmental interference and avoids the characteristics of maize disease spots in the high and low frequency images. Second, the high and low frequency multi-channel multi-scale fusion network structure (MARNet) with attenuation factor is established, which improves the model feature extraction ability while enhancing the robustness of the deep network. Finally, PRelu and adabound are selected as the activation functions and optimizers of the WG-MARNet. The structure of the WG-MARNet is shown in Fig 2.

The input image size used in this network is a three-channel image of 224×224. The image is decomposed into high and low frequencies using WT-GBF to obtain low-frequency images and high-frequency images. Then the high-frequency images are taken again to obtain the weakened background information. At the same time, the higher frequency image that enhances the lesion information, the low-frequency image and the higher frequency image are fed into the network in a channel. The following analysis of the network takes the low-frequency channel as an example.

The first layer of the network is the convolution layer, which contains 64 channel convolution operations. Then, the batch normalization layer is used to re parameterize the distribution of feature maps. Then, a nonlinear excitation layer PRelu is added to introduce nonlinearity into the network of this layer. Every two network feature extraction layers need to be added with a nonlinear excitation function to introduce nonlinearity. Otherwise, multiple feature extraction network layers can be represented by one feature extraction network layer, which cannot introduce stronger feature extraction ability and wasted computing resources. The reason why residual structure is not used directly from the first convolution layer is that the feature map is used instead of the original input image when a shortcut is directly connected. The following feature extraction network is divided into 16 residual blocks with 4 groups, conv2-x to conv5-x. Each residual block group conv-x contains a corresponding number of residual blocks. Table 3 shows that the number, size, and step size of convolution kernels in each residual block are also included in the table.

**Table 3:** Parameter configuration of residual block in conv1-conv5 group.

Conv1	Conv2_x	Conv3_x	Conv4_x	Conv5_x
7×7,64, 3×3max pool, stride2	$[\begin{array}{l} 1\times 1,64\\ 3\times 3,64\\ 1\times 1,256 \end{array}]\times 3$	$[\begin{array}{l} 1\times 1,128\\ 3\times 3,128\\ 1\times 1,512 \end{array}]\times 4$	$[\begin{array}{l} 1\times 1,256\\ 3\times 3,256\\ 1\times 1,1024 \end{array}]\times 6$	$[\begin{array}{l} 1\times 1,512\\ 3\times 3,512\\ 1\times 1,2048 \end{array}]\times 3$

**Fig 3:**
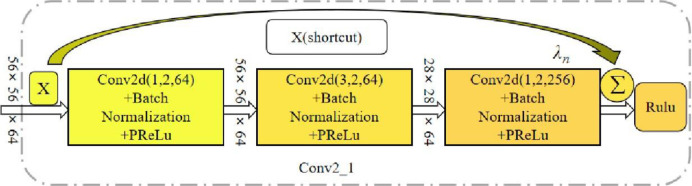
Conv2_ 1 parameter operation diagram.

The block diagram of a residual block network in conv2_x residual group is given below, as shown in Fig 3.

The picture above is the structure of the first residual block Conv2_1 in the Conv2_X residual group. After Conv1_X convolution, the input feature map is a 64-channel 56×56 feature map. After three convolution kernels extract features, the output becomes a 28×28 feature map with 64 channels. The parameter "3" in the figure indicates that the size of the convolution kernel is 3×3, "2" indicates that the stride of the convolution layer is 2, and "64" indicates the output channel. The value of the stride determines whether the size of the output feature map will change. When stride is 2, it means that the size of the output feature map is half of the input feature map, and the shortcut connection part is for a convolution operation to make the output feature map. The number of channels is the same as that of the shortcut so that the connection operation of Element wise Add can be performed.

In addition, dropout is added to the fully connected layer to prevent overfitting further. Among them, after average pooling, the sizes of the output characteristic graphs of conv2_x, conv3_x, conv4_x, and conv5_x are changed into [56,56256], [28,28512], [14,141024], [7,7, 2048], if conv2_x, conv3_x, and conv5_x feature vectors are selected for multi-feature fusion, then the fused feature vectors are spliced into a 2816 dimensional feature vector, and then the high and low-frequency feature vectors are fused to generate a 5632-dimensional feature vector for the following network classification.

#### 3.2.1 Denoising and high and low frequency decomposition of maize leaf disease images

Generally speaking, due to the influence of image acquisition equipment and shooting conditions, there is always some noise in maize images. The goal of WT-GBF processing is to reduce the impact of these noises (small-scale texture details, outliers and spots, etc.), highlight useful information (object edges, foreground, and background boundaries, etc.) to improve the accuracy of subsequent network recognition. At the same time, due to the characteristics of maize diseases (Large spot disease, small spot disease, and rust, etc.), the leaf disease information is mainly manifested in the high-frequency part of the image, and only a small part of the disease information remains in the low-frequency background image. WT-GBF decomposes high and low-frequency images to realize subsequent network sub-channel processing of high and low-frequency images and improve feature extraction capabilities.

**Fig 4:**
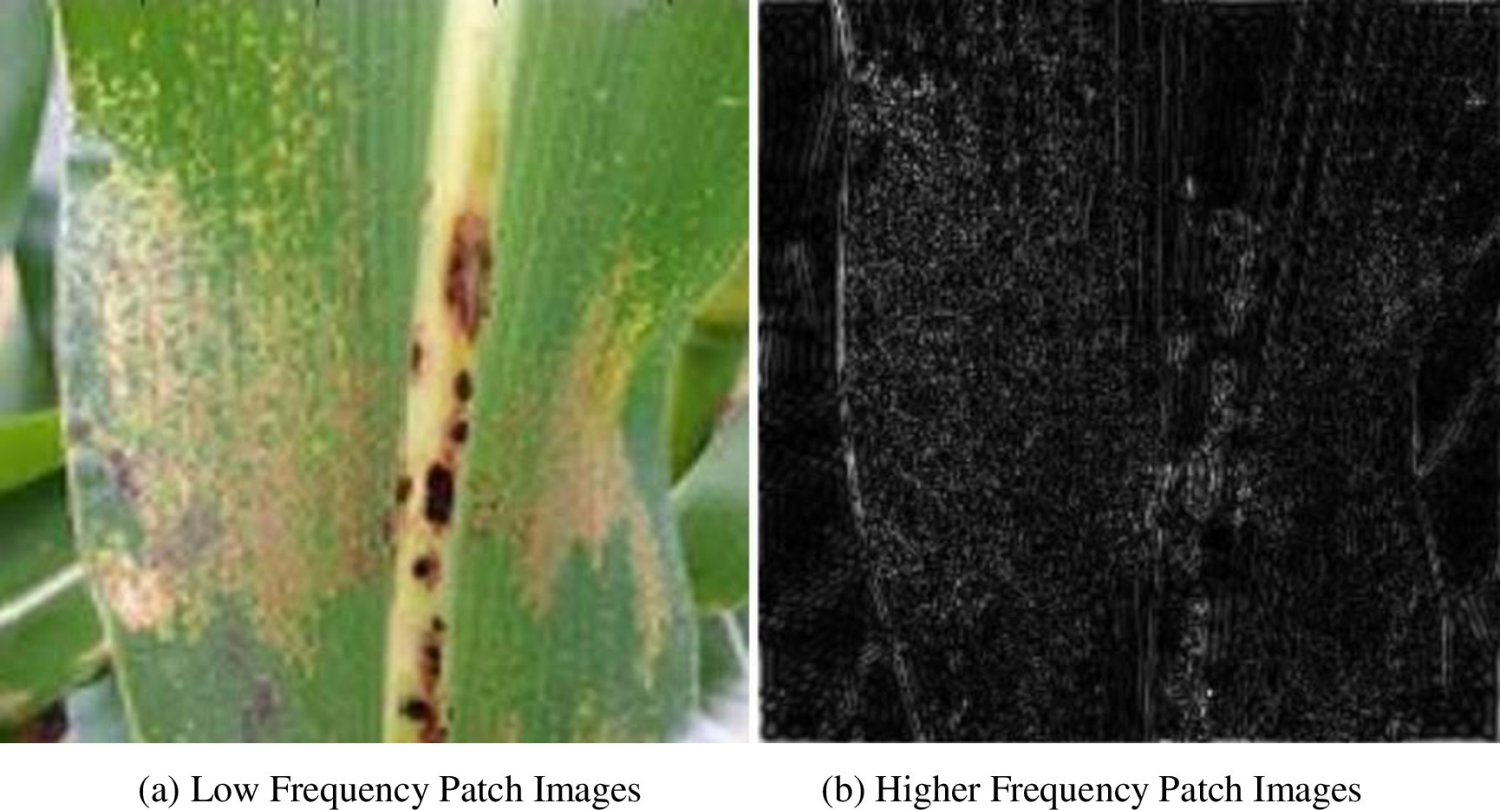
High and low frequency decomposition effect picture. (a) Low Frequency Patch Images. (b) Higher Frequency Patch Images.

The WT-GBF is chosen to decompose the high and low frequencies of the image. The reason is that compared with other commonly used filters, this algorithm can well retain the detailed texture of the low-frequency background image and has the effect of maintaining the boundary. The two kernel functions in bilateral filtering are combined spatial domain function and range kernel function. It is precise because of the role of these two kernel functions in the filtering process that bilateral filtering has edge and detail retention characteristics. The effect of wavelet threshold denoising is very good. Firstly, the maize disease image is denoised by the wavelet threshold to obtain a smoother image. The wavelet threshold uses a hard threshold, and then the smoothed image is used as a guide image for calculating the kernel function of bilateral filtering. Fig 4 shows the comparison of several common filters. It can be seen that the low-frequency image obtained by using wavelet threshold to guide bilateral filtering has better detail retention.

After the maize lesion image is subjected to WT-GBF, the low-frequency background image *I*_*L*_ and the high-frequency lesion image *I*_*H*_ are obtained. In order to obtain a higher frequency image *I*_*HH*_, the high frequency image *I*_*H*_ needs to be passed through the filter again, which can be expressed as:

$$\left\{ \begin{array}{l} I=I_{L}+I_{H}\\ I_{H}=I_{HL}+I_{HH} \end{array}\right.$$ (1)

The images of maize disease images decomposed by high and low frequencies are shown in Fig 4.

#### 3.2.2 Multiscale feature fusion

With the training of deep neural networks, the content of the features extracted by the network will also vary greatly with the different feature levels [28], and these levels of information have their own characteristics. For image tasks: In general, the features extracted by the shallow network contain rich detailed information on the image content. However, due to the relatively shallow feature level, there will be a lot of redundant information in the information contained. If the information is directly used for classification, the effect is often unsatisfactory due to the lack of high-level semantic information; the information extracted from the deep network contains more semantic information, and compared with the features extracted from the shallow network, there is no Too much detailed information of the image content. The positioning information is not accurate enough. Because the extracted information is too refined and too abstract, it also leads to the lack of information to some extent, and the integrity of the information cannot be guaranteed. The hierarchical features are between the shallow features and the deep features. There is a certain amount of detailed information on the image content, as well as high-level semantic information, and the information content is relatively complete.

**Fig 5:**
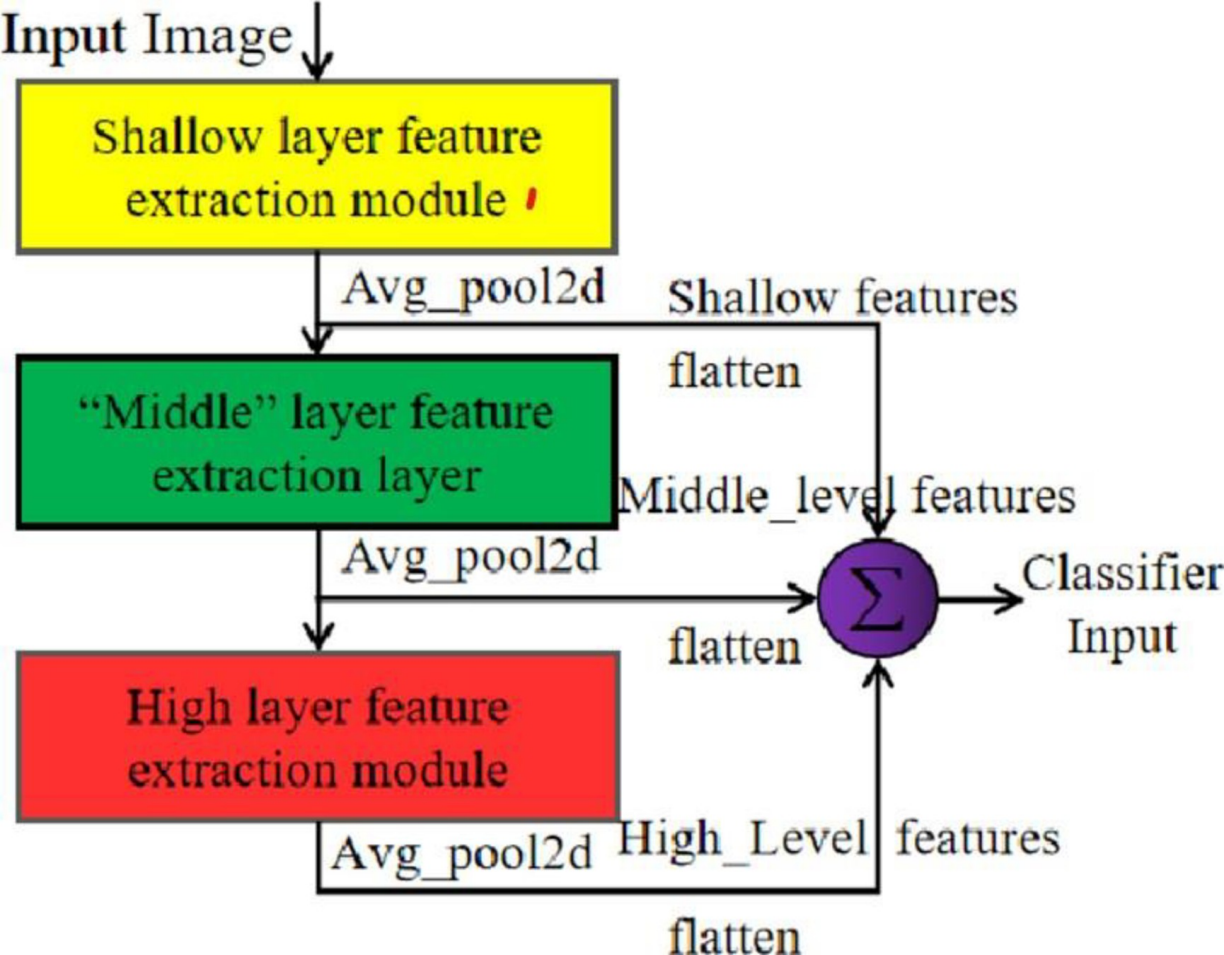
Multi-scale feature fusion method based on task design.

In the feature fusion method shown in Fig 5, we first average down-sampling the extracted shallow, middle and high-level features to reduce the size of the feature map and the number of features. Then we tile the features, stitch the features of each level after tiling to get a fused feature vector, which will function as the last feature vector. The result is predicted by the training classifier. This method eliminates the operation required for progressive fusion and reduces the number of features. Finally, the three features are fused directly for classification. Compared with the existing popular methods, the fusion method proposed in this paper is more suitable for this research task.

#### 3.2.3 Multi-channel feature fusion

**Fig 6:**
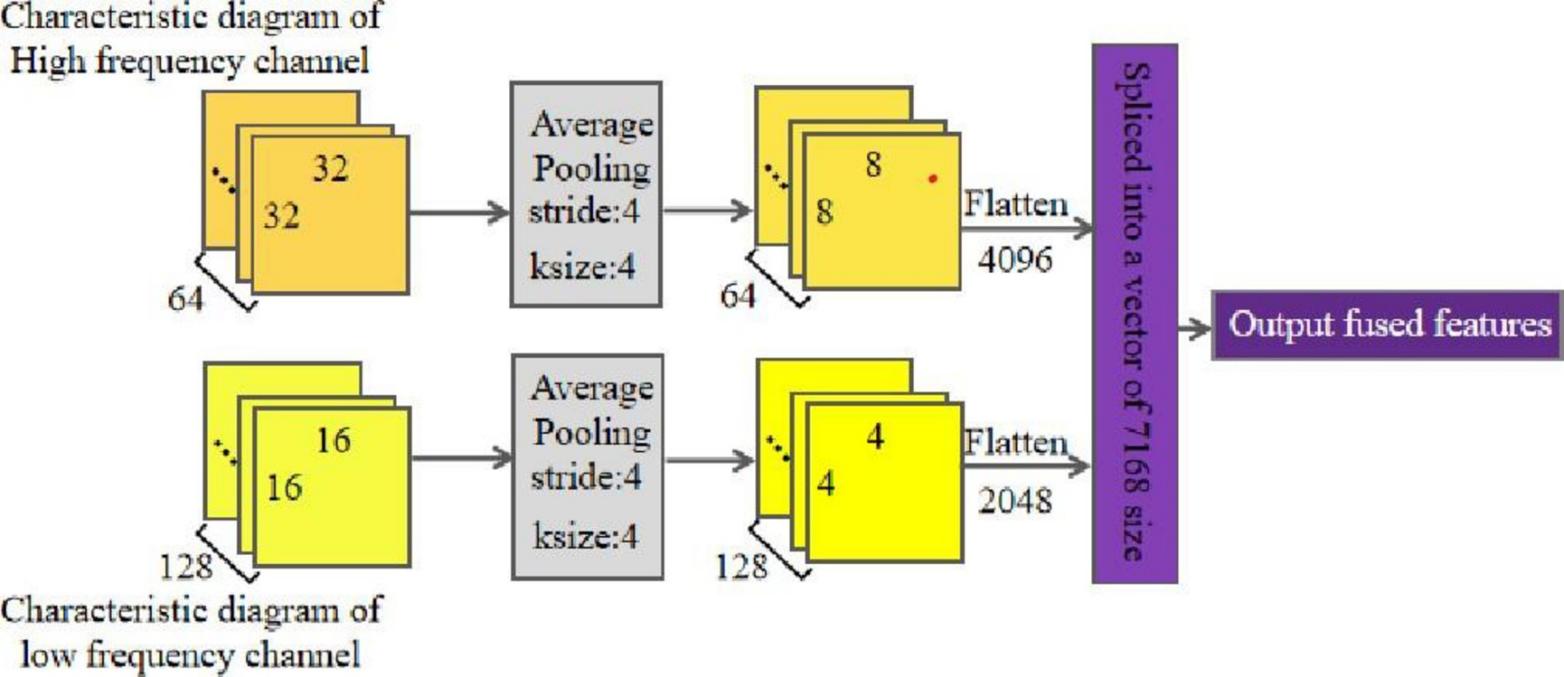
Multi-channel feature fusion process diagram.

The feature fusion layer of a multi-channel convolution neural network can fuse different feature information, which makes the fused feature information more distinguishable and better expressive ability for images. After the input image is pooled by convolution of each channel in the multi-channel convolution neural network, the output feature maps of the high-frequency channel and the low-frequency channel can be obtained, respectively. Before feature fusion, Average pooling was performed on the feature map. A kernel size of 4 and a stride of 4 were used to reduce the dimensions of the extracted features. Then the two-dimensional picture data is transformed into one-dimensional feature vectors, and the data is batch standardized to make the data distribution more dispersed and closer to the data distribution of the test set, which reduces the model overfitting. Finally, the processed one-dimensional data is input into the full connection layer for feature information fusion. The multi-channel feature fusion process is shown in Fig 6.

#### 3.2.4 Attenuation factor

**Fig 7:**
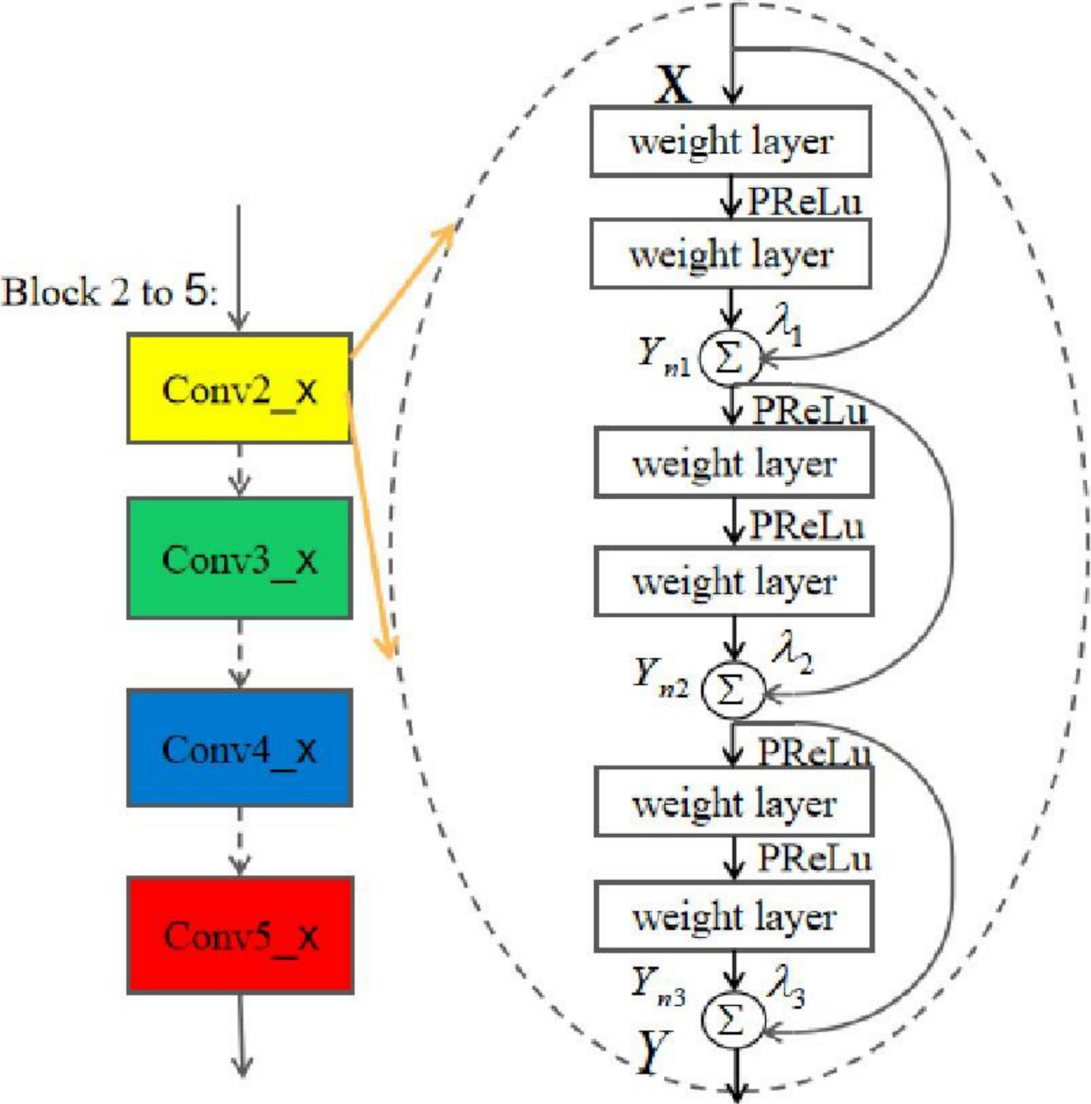
Attenuation factor introduction graph.

To increase the stability of the learning process of neural network parameters, a convolution attenuation factor is introduced into the convolution channel, and the sparse restriction on the output characteristic graph of each convolution module is applied [29]. The data flow on each channel of WG-MARNet is controlled and managed. The structure block scheme shown in Fig 7 is adopted in this paper.

The nonlinear mapping function for a single structure is:

$$Y=\lambda_{1}Y_{n1}+\lambda_{2}Y_{n2}+\lambda_{3}Y_{n3}X$$ (2)

In the expression, Σ means summing up the neurons in the corresponding output characteristic map of each channel. *λ*_1_, *λ*_2_, and *λ*_3_ are convolution decay factors, and *0*<*λ*_3_<*λ*_2_<*λ*_1_≤1. The convolution module shares a convolution attenuation factor with all the neurons on the output characteristic graph. Different convolution modules will use different size attenuation factors.

Formula (2) shows that the output of WG-MARNet is determined by the output data of each channel. Based on the contribution of each channel to the output of the neural network, a concept of network output contribution ratio is introduced to define the contribution ratio of network output to the attenuation factor of each channel. The Shortcut channel has no attenuation factor, so its attenuation factor is equivalent to 1, so the contribution ratio of neural network output to the Shortcut channel *M* = 1/(1+*λ*_1_+*λ*_2_+*λ*_3_). The attenuation factors of the three convolution modules on the convolution channel are *λ*_1_, *λ*_2_, and *λ*_3_ which are artificially set network hyperparameters, so their network output contribution rates are:

$$\left\{ \begin{array}{l} M_{n1}=\lambda_{1}/(1+\lambda_{1}+\lambda_{2}+\lambda_{3})\\ M_{n2}=\lambda_{2}/(1+\lambda_{1}+\lambda_{2}+\lambda_{3})\\ M_{n3}=\lambda_{3}/(1+\lambda_{1}+\lambda_{2}+\lambda_{3}) \end{array}\right.$$ (3)

Because 0<*λ*_3_<*λ*_2_<*λ*_1_≤1, there is *M*_*n*3_<*M*_n2_<*M*_n1_<*M*. From the perspective of forward propagation, the attenuation factor in this paper is designed as follows: The final output of the network is dominated by convolution channels, supplemented by the Shortcut channels, dominated by shallow features and supplemented by deep features. Therefore, the range of attenuation factor is limited to *0*<*λ*_3_<*λ*_2_<*λ*_1_≤1, which is based on the following: First, the convolution channel is always changing due to the training parameters, and the output of the front layer network will be used as the input of the back layer network. The effect of the change of the front layer network parameters on the subsequent output results will increase exponentially with the increase of network depth, resulting in the deep network being prone to a local optimum. Solutions are difficult to converge, so to enhance the stability of the network, the attenuation factor is set to less than 1. secondly, from the perspective of a single structure block, the Shortcut channel is equivalent to data passing through 0 convolution layers for feature extraction, the first convolution module is equivalent to data passing through 2 convolution layers, the second convolution module is equivalent to data passing through 4 convolution layers. In order to ensure that the output contribution rate should be dominated by shallow features and supplemented by deep features, so *λ*_3_<*λ*_2_<*λ*_1_.

From the point of view of backward propagation, the convolution attenuation factor can control the size of the gradient values propagating from back to forward. The attenuation factor plays a role in reducing the learning rate of weights for each layer of the network, so that the weights parameters can be adjusted more finely during network training. And it also helps to improve the robustness of the network.

## 4. Application and results analysis

### 4.1 Experiments setup

The experimental platform chooses Windows10 operating system, equipped with Core i7-7770KCPU@4.00GHX8 processor and NvidiaGeforceGTX1080TiGPU.

We employ stochastic gradient descent (SGD) while considering the performance of hardware devices and training effects. The batchsize during training is set to 32, the momentum parameter is set to 0.9, and the number of epochs is set to 15. The AdaBound optimizer is used in this paper’s model. Because changing the learning rate affects the model’s convergence speed and stability, a callback function is included. The learning rate is set to 0.01 for the first 10 epochs, and the weight decay rate is set to 0.05 for the next 5 epochs to boost fitting speed.

### 4.2 Experimental evaluation index

In order to evaluate the test results of the proposed method for disease identification and classification, the precision and recall were calculated respectively after the samples were tested. The comprehensive evaluation index *F*1 is used as the evaluation value of accuracy and recall rate. Average accuracy (AA) and single image detection time (t) were introduced as evaluation indexes. AA = number of correct classification / total number of tests × 100%, t = total test time / number of test images. Other indicators are calculated as follows:

$$P=\frac{\mathrm{TP}}{\mathrm{TP}+\mathrm{FP}}$$ (4)

$$R=\frac{\mathrm{TP}}{\mathrm{TP}+\mathrm{FN}}$$ (5)

$$F1=\frac{2\times P\times R}{P+R}$$ (6)

P represents the precision, while R represents the recall rate. TP represents the number of samples that are actually targets, and the model predicts that the sample is a target (detecting a positive sample as a positive sample). In Eq 8, FP represents the number of samples that are not actually targets, but the model predicts that the sample is a target (tests negative samples as positive samples). In Eq 9, FN represents the number of samples that are actually targets, but the model did not predict them as target (no positive samples were detected as positive samples).

### 4.3 Performance and analysis

#### 4.3.1 Recognition results and confusion matrix

**Fig 8:**
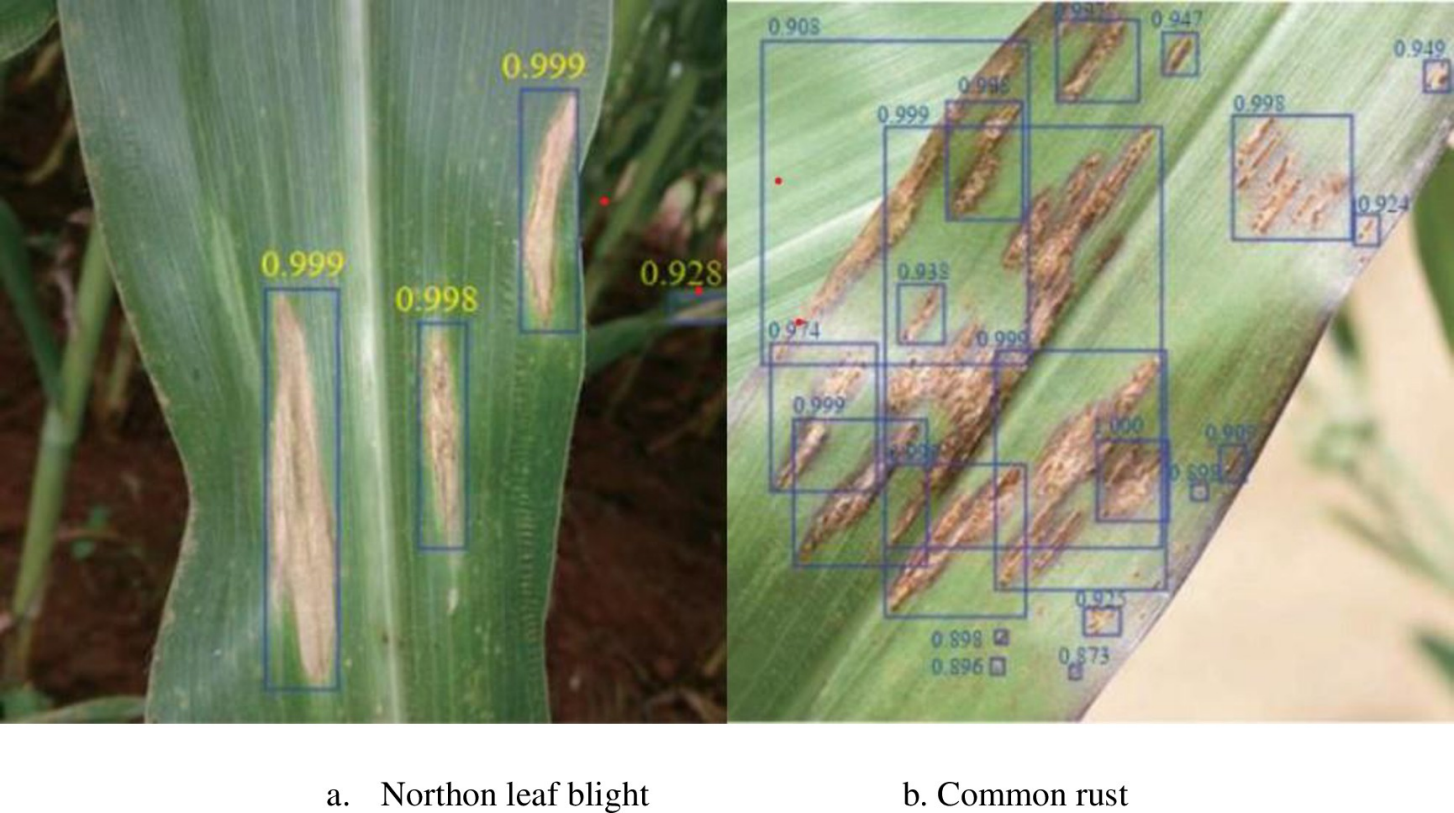
Example of recognition results of northern leaf blight and common rust. a. Northon leaf blight. b. Common rust.

**Fig 9:**
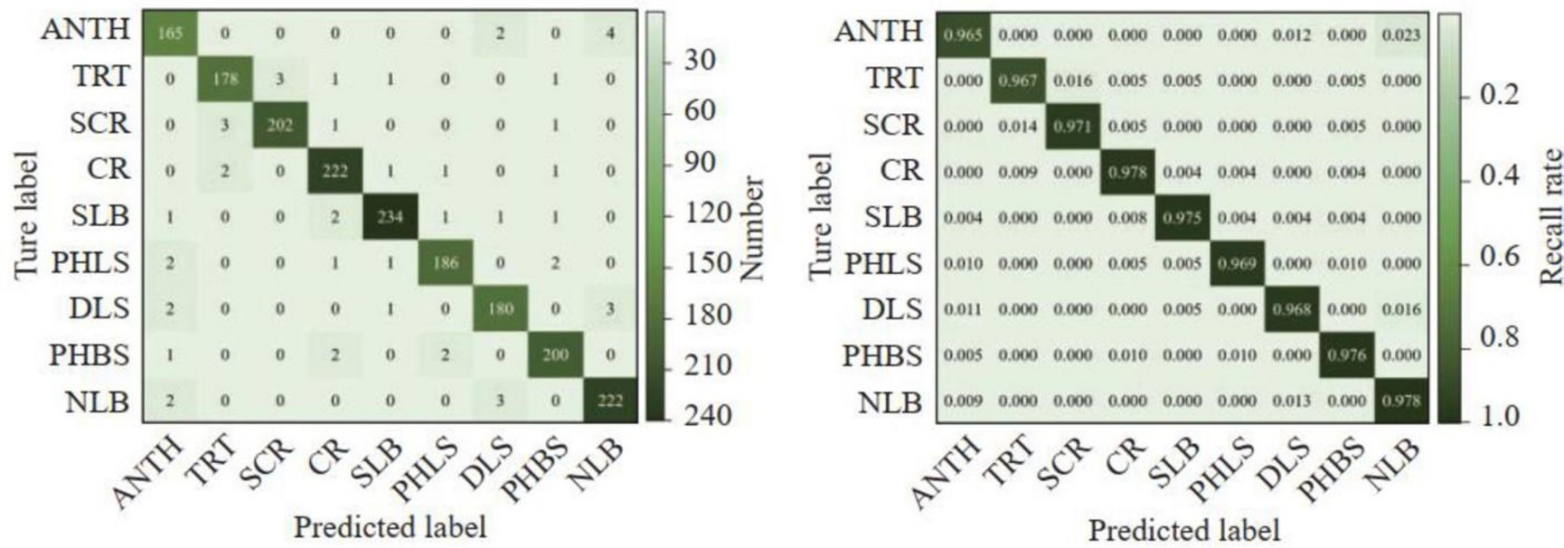
ANTH, TRT, SCR, CR, SLB, PHLS, DLS, PHRS and NLB represent Anthracnose leaf blight, Tropical rust, Southern maizerust, Common rust, Southern leaf blight, Phaeosp haeria leaf spot, Diplodia leaf streak, Physoderma brown spot and Northern leaf blight respectively; The darker diagonal values in figures represent the number of correct classifications and the recall rate of each category, respectively.

Taking northern leaf blight disease and common rust disease as examples, Fig 8 shows the recognition results of WG-MARNet in the test set. It can be found that the proposed method can accurately locate the lesion using the positioning frame and output the recognition probability. The disease spots can also be accurately identified and located under the background interference of maize stalks and soil (Fig 8A). It can be seen from Fig 8 that WG-MARNet can achieve high accuracy for image recognition under different lighting conditions, including maize stalks, soil, and overlapping leaves (The recognition probability of most lesions is high. At 0.9). In order to show the recognition accuracy and classification results of WG-MARNet more clearly, a confusion matrix is drawn based on the model classification results on the test set (Fig 9). Combined with the confusion matrix analysis of the disease recognition results, the proposed method is suitable for 9 different types of Anthracnose leaf blight, Tropical rust, Southern maize rust, Common rust, Southern leaf blight, Phaeosp haeria leaf spot, Diplodia leaf streak, Physoderma brown spot, and Northern leaf blight. When identifying disease types, the accuracy and recall rate of each disease type are different. This has a certain relationship with the feature type of each disease, but the recognition accuracy of each disease type is maintained at 0.9682~0.9897, the average accuracy is 0.9762; the recall rate is maintained at 0.954~0.983, the average recall rate is 0.9719. F1 is 0.959 ~0.979, the average accuracy of the model is 97.43%. The above results show that the proposed method performs well in the established data set and can be applied to the detection of crop diseases in the actual field environment.

#### 4.3.2 Effect of multi-scale feature fusion on model performance

**Fig 10:**
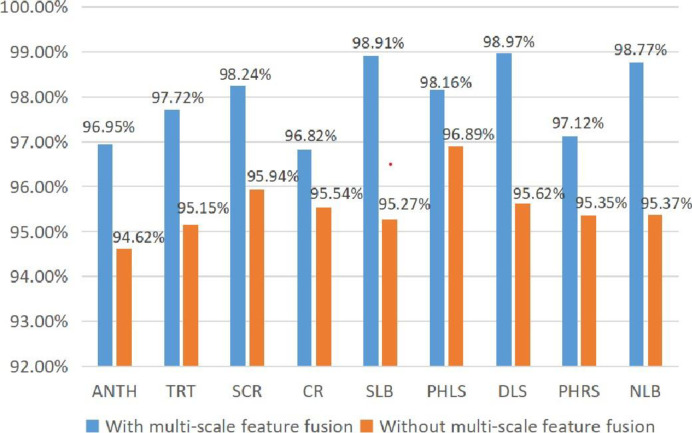
Effect comparison chart of multi-scale feature fusion.

To analyze the impact of the multi-scale feature fusion algorithm on model performance, this part of the experiment used 80% of the datasets to train the WG-MARNet models with and without multi-scale feature fusion and then validated them with 20% of the datasets. The results of the experiment are shown in Fig 10. After applying the multi-scale feature fusion algorithm, the maximum accuracy can be increased to 98.97%. And the minimum accuracy can be improved to 96.95%.

Under the same conditions, the accuracy of nine disease images was improved by 2.33%, 2.57%, 2.30%, 1.28%, 3.64%, 1.27%, 3.35%, 1.77% and 3.40%, respectively. The recognition accuracy has been significantly improved. This shows that because the information extracted by the deep network is too refined and too abstract, the information is missing to a certain extent. Therefore, our multi-scale feature fusion algorithm effectively improves the recognition accuracy in practice.

#### 4.3.3 Comparative experiments of different activation functions and optimizers

In the construction of the ResNet network, Relu or Sigmoid is used as the activation function, and Adam is the optimizer as the mainstream choice. We have chosen activation functions (Relu, Sigmoid, PRelu) and optimizers (Adam, Adabound) to form multiple solutions. The scheme configuration is shown in Table 4. The first part compares the choice of optimizers on the premise of a unified activation function. The experiment includes the comparison between Sheme1 and Sheme6, the comparison between Sheme2 and Sheme5, and the comparison between Sheme3 and Sheme4. It can be seen from Table 4 that although the *F*1 of Scheme 1 is 0.51% higher than the *F*1 of Scheme 6, the Loss is higher than that of Scheme 6. Other programs 4 and 5 that use Adabound perform better than those that use Adam. The other part compares the choice of activation function under the premise of a unified optimizer. The experiment included a comparison between 1-2-3 and 4-5-6. Scheme 3 using the Prelu function has lower Loss than schemes 1 and 2; *F*1 and Loss of Scheme 5 and Scheme 6 are lower than Scheme 1; *F*1 of Scheme 4 reached 97.89% while Loss was close to 0. Based on the experimental results and analysis, we finally decided to use Adabound as the optimizer and PRelu as the activation function.

**Table 4:** Scheme configuration of optimizer and activation function.

Schemes	Optimizer	Activation Function	Loss	*F*_1_(%)
Sheme1	Adam	Relu	0.041	97.14
Sheme2	Adam	Sigmoid	0.037	96.35
Sheme3	Adam	PRelu	0.027	97.17
Sheme4	AdaBound	PRelu	0.012	97.89
Sheme5	AdaBound	Relu	0.035	96.63
Sheme6	AdaBound	Sigmoid	0.029	96.71

#### 4.3.4 Test results compared with other classification algorithms

**Fig 11:**
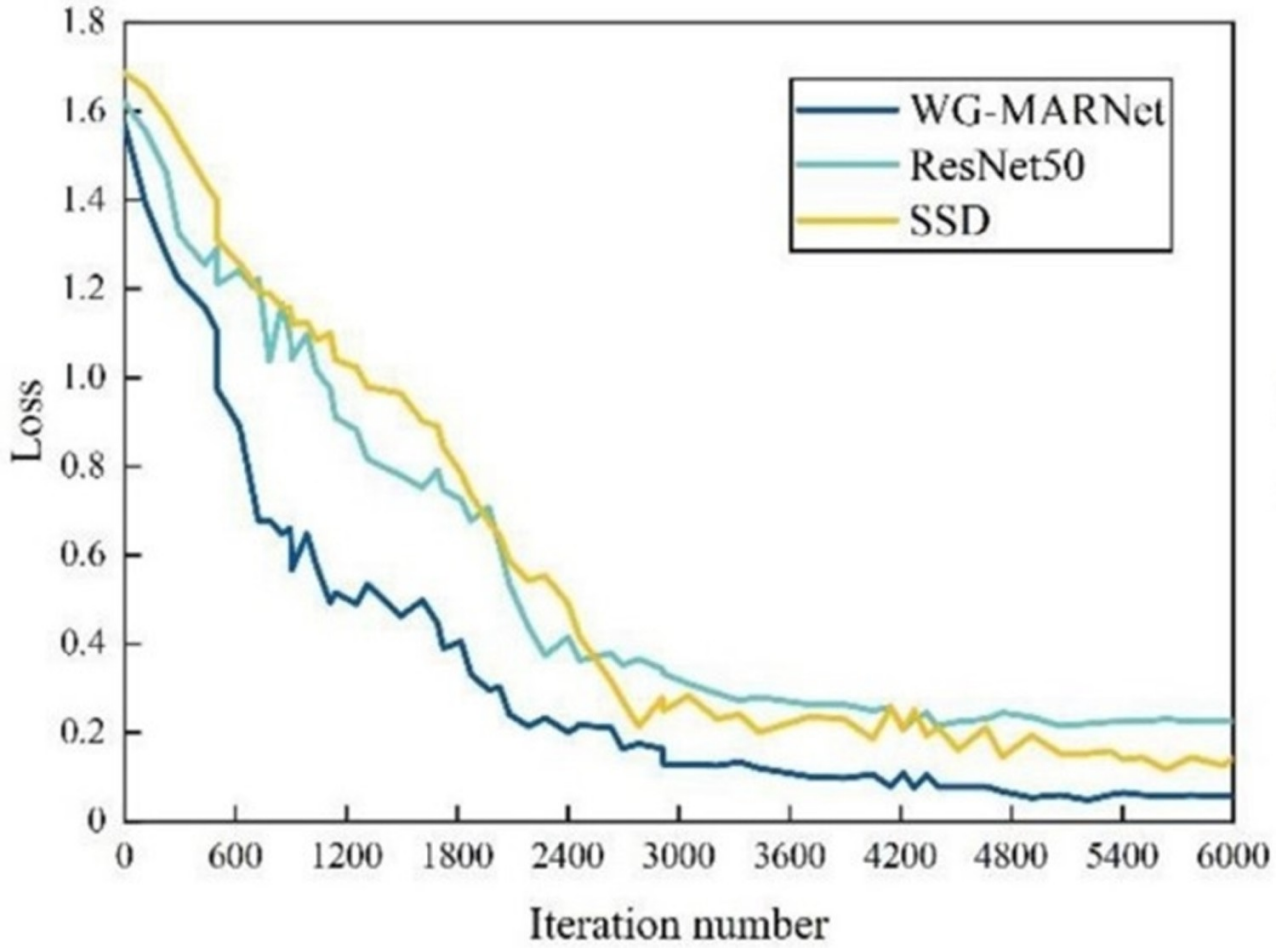
The loss curves of different classification and detection algorithms varying with the iteration number.

**Fig 12:**
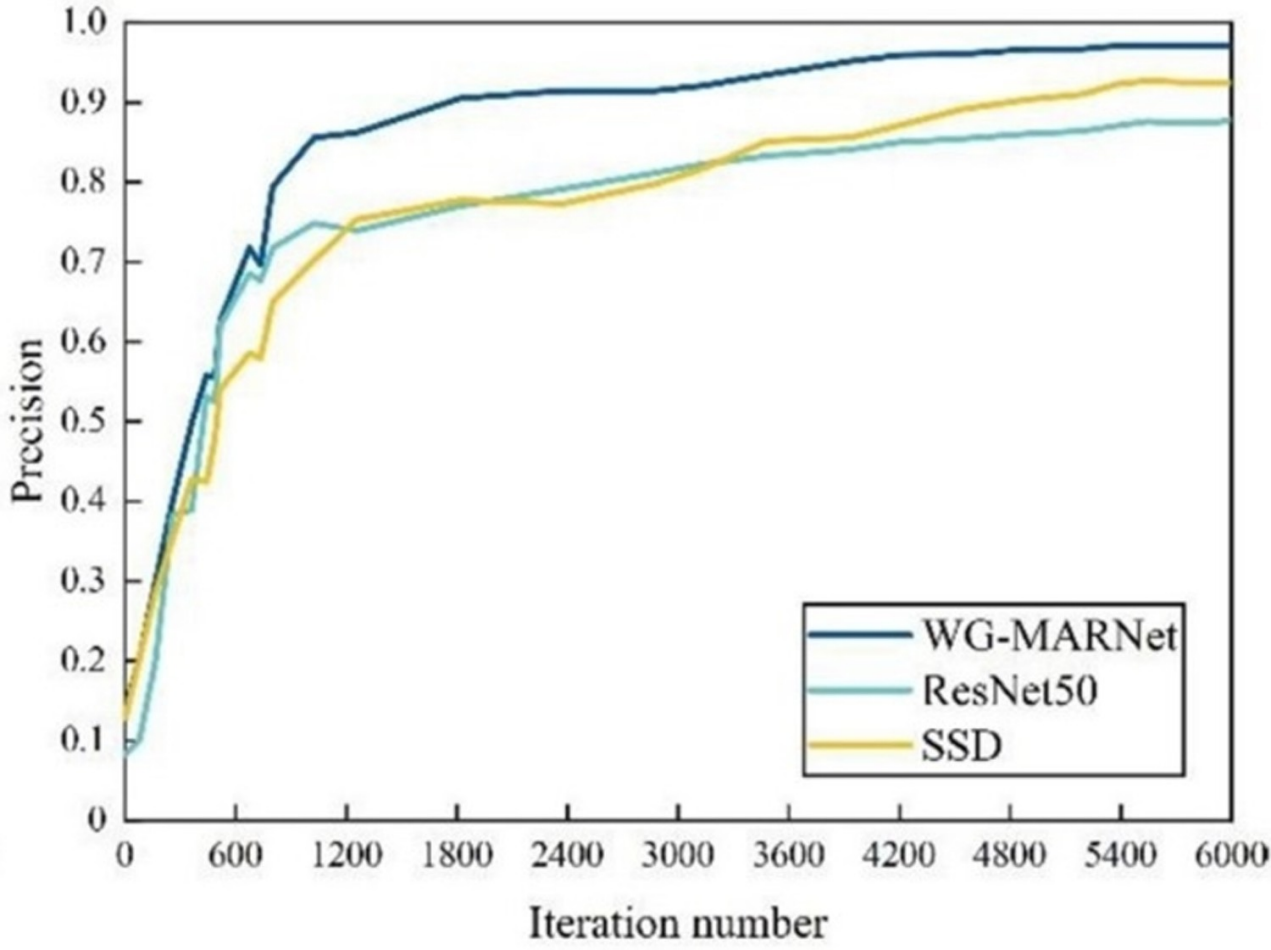
The precision curves of different classification and detection algorithms varying with the iteration number.

To further verify the performance of WG-MARNet proposed in this paper, it is compared with RstNet50 and the main target detection Single Shot Multi-Box Detector (SSD) algorithms. SSD uses a 16-layer VGG feature extraction network. The three methods use the same dataset, and the training methods are all Stochastic Gradient Descent (SGD). The initial learning rate of the three methods is 0.01, the dropout value is 0.6, and the maximum number of iterations is 6000. Finally, the curve charts of loss values with the number of iterations for the three classification detection algorithms are obtained (Fig 11), and the curve charts of accuracy with the number of iterations (Fig 12). Table 6 shows the average accuracy and time-consuming of the three methods.

We assume that the WG-MARNet performs better than other networks. The null hypothesis is that the average accuracy of the WG-MARNet is the same as the average accuracy of other networks in inferential statistics. We repeatedly measured the accuracy of the experiment ten times, conducting a one-way analysis of variance with α = 0.05. As shown in Table 5, the P value is 2.44 × 10^−4^, which is much smaller than α, and the F value is 8.815, which is greater than the critical value of 2.606. The results mean that the null hypothesis is rejected. The analysis demonstrates a significant difference in accuracy between the WG-MARNet and other networks.

**Table 5:** Comparison of the detection results for different classification and detection algorithms.

Module	Precision	F	p-value	F Crit	Detection time per /s
SSD	0.9293	8.815	2.44×10^−4^	2.606	0.293
ResNet50	0.8832				0.417
WG-MARNet	0.9718				0.278
SCANet [30]	0.9223				0.325
reAlexNet [24]	0.9245				0.368

The loss value of all three methods lowers as the number of repetitions grows, with the loss value decreasing the fastest at the start due to the high initial learning rate setting. WG-MARNet has the lowest loss value of the three and achieves convergence the quickest. According to Table 5, WG-MARNet has the lowest loss value among the five, and the proposed algorithm achieves an accuracy of 0.9718 and a single image detection time of 0.278s. In comparison to the new algorithms SCANet [27], Re Alex Net [28], the WG-MARNet algorithm proposed in this paper improves the multi-scale feature fusion technique and introduces attenuation factors on the channels to identify maize disease images more accurately and efficiently, which has some advantages. The algorithm’s accuracy is increased by 4.95% and 4.73%, respectively, while the detection time of a single image is lowered by 0.047 seconds and 0.090 seconds. In terms of detecting speed and accuracy, the experimental results reveal that WG-MARNet surpasses the original ResNet50, SSD. And the new SCANet, Re Alex Net method, have improved overall performance and can be employed in the field for real-time detection of maize diseases in complicated backgrounds.

#### 4.3.5 Ablation experiments

Experiments were chosen to design ablation experiments utilizing an 80% data set to evaluate the impact of each component of the proposed method on the performance of the WG-MARNet. The ResNet network was chosen as the base network structure to assess the performance impact of the Denoising and High and Low-frequency Decomposition module (DHLD), the Multi-scale Feature Fusion module (MFF), the Multi-channel Feature Fusion module (MCFF), and the Addition of the attenuation Factor module (AF) on the WG-MARNet model. When training the deep network, the attenuation factor is applied to both high and low-frequency multi-channels to reduce performance instability.

According to the data in Table 6, the basic network structure Resnet (network (1)) achieved an average precision of 91.77% and an average recall rate of 92.53% on this dataset, and the model’s average accuracy was 92.22%. Denoising and high and low-frequency decomposition result in average precision and recall rates of 92.58% and 93.46%, respectively, and the created high and low-frequency multi-channel network structure considerably improves feature extraction capability. The network (3)’s multi-scale feature fusion methodology was improved by applying the average downsampling and tiling method to boost target feature expression capability. The addition of the multi-scale feature fusion module structure based on denoising and high and low-frequency decomposition greatly improves model performance. The evaluation indices improve by 1.20%, 1.38%, and 1.61%, respectively, compared to a network (2). In comparison to network (3), network (4) includes a multi-channel feature fusion module to fuse disparate feature information and increase differentiation and picture representation capability. When compared to a network (3), it improves by 1.26%, 1.04%, and 0.17% in each evaluation index. Finally, the WG-MARNet (network (5)) incorporates attenuation factors on both high-frequency and low-frequency channels to enhance the unstable performance when training the deep network, allowing the weight parameters to be fine-tuned during training improving the network’s robustness. According to the experimental results, the addition of attenuation factors on high and low-frequency channels reaches 97.62%, 97.19%, and 97.43% for each evaluation index. The experimental results reveal that each component significantly improves the WG-MARNet’s performance.

**Table 6:** Experimental results of each component of the WG-MARNet.

Network	Accuracy	Recall	F_1_(%)
ResNet (1)	88.32%	87.95%	89.12%
ResNet+DHLD (2)	92.58%	93.46%	93.33%
ResNet+DHLD+			
MFF (3)	93.78%	94.84%	94.94%
ResNet+DHLD+			
MFF+MCFF (4)	95.04%	95.88%	95.11%
ResNet+DHLD+			
MFF+MCFF+AF			
WG-MARNet(WG-MARNet) (5)	97.62%	97.19%	97.43%

## 5. Conclusion

In this paper, we propose an Internet of Things (IoT) maize leaf disease identification method based on WG-MARNet. To begin, the ResNet50 network is expanded to multiple channels, and the low- and high-frequency images are sent into separate channels. The multi-scale feature fusion technique is then enhanced by mean down-sampling and tiling techniques. To improve the target feature expression, the multi-scale feature fusion technique is utilized; an attenuation factor is added to the channels to solve the problem that the deep network is prone to unstable performance during training. After using the PRelu activation function and the AdaBound optimizer, our network can achieve an accuracy of up to 97.89% with a Loss of 0.012. Combined with the confusion matrix analysis of the disease identification results, the proposed method has an average recall of 97.19% and an F1 of 95.9% to 97.9% when performing classification, with an average accuracy of 97.43% for the model. The experimental results demonstrate the efficacy of the proposed maize leaf disease detection algorithm, which achieves an accuracy of 0.9718 and a single image detection time of 0.278s when compared to contemporary techniques ResNet50, SSD, SCANet, and Re Alex Net.

This paper can provide methods and concepts for intelligent monitoring of maize diseases in the field environment, as well as lay the groundwork for early and accurate maize disease prevention and control. Furthermore, based on the findings of this study, it can serve as a foundation for additional field-wide disease patrol monitoring using continuous frame video. Despite the proposed method’s high performance, the data used in this experiment are limited. The data set is not particularly large for WG-MARNet, so the image data set will be increased in the future to develop a more representative and sample feature-rich maize disease database.

## Supporting information

S1 File (ZIP)
