# Supplementary figures and images for: Maize leaf disease identification based on WG-MARNet (part 2 of 2)
Source: PLoS One. 2022 Apr 28;17(4):e0267650. doi: 10.1371/journal.pone.0267650 (PMC9050012; doi:10.1371/journal.pone.0267650)

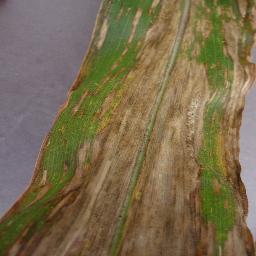

Supplement: S1 File — (ZIP) [file pone.0267650.s001.zip › All data sets/Anthracnose leaf blight/fd9c7938-4f3f-448d-851b-042e287515f0___RS_NLB3990.JPG]

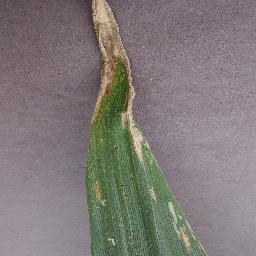

Supplement: S1 File — (ZIP) [file pone.0267650.s001.zip › All data sets/Anthracnose leaf blight/fda22c64-49ee-4863-a94e-6e16ca177099___RS_NLB3798.JPG]

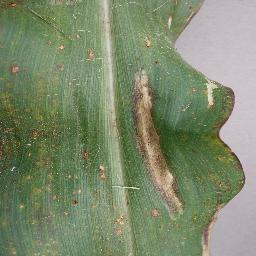

Supplement: S1 File — (ZIP) [file pone.0267650.s001.zip › All data sets/Anthracnose leaf blight/fdff294e-df6e-42eb-9bcf-edecf4002891___RS_NLB4158.JPG]

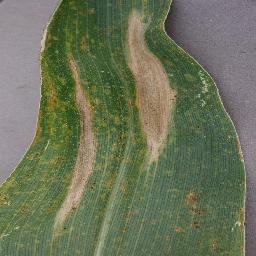

Supplement: S1 File — (ZIP) [file pone.0267650.s001.zip › All data sets/Anthracnose leaf blight/fe2aa542-8ace-4e0d-8315-48d4c6d023ef___RS_NLB4271.JPG]

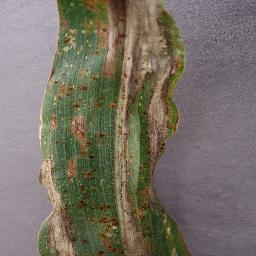

Supplement: S1 File — (ZIP) [file pone.0267650.s001.zip › All data sets/Anthracnose leaf blight/fed381d0-4407-4ec5-a178-d70ec1ca93d3___RS_NLB3914.JPG]

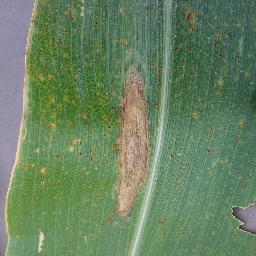

Supplement: S1 File — (ZIP) [file pone.0267650.s001.zip › All data sets/Anthracnose leaf blight/ff8577c3-ad10-4272-84f6-b7c6d6042d1f___RS_NLB3506.JPG]

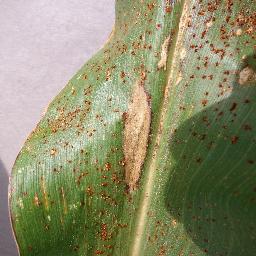

Supplement: S1 File — (ZIP) [file pone.0267650.s001.zip › All data sets/Anthracnose leaf blight/ffa82f14-8320-469b-a621-6475c92a4bb8___RS_NLB3649.JPG]

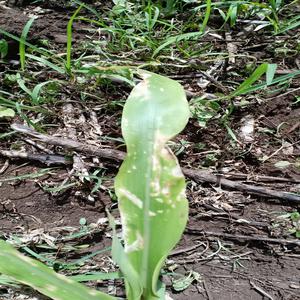

Supplement: S1 File — (ZIP) [file pone.0267650.s001.zip › All data sets/Common rust/IMG20190523113835.jpg]

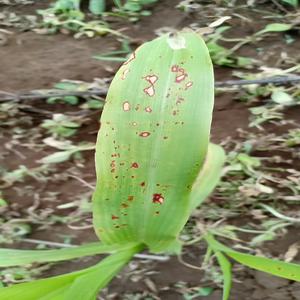

Supplement: S1 File — (ZIP) [file pone.0267650.s001.zip › All data sets/Common rust/IMG20190523113901.jpg]

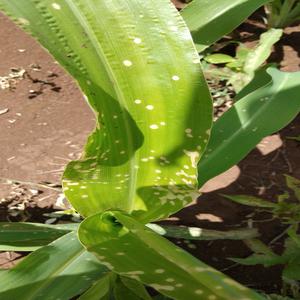

Supplement: S1 File — (ZIP) [file pone.0267650.s001.zip › All data sets/Common rust/IMG20190523115007.jpg]

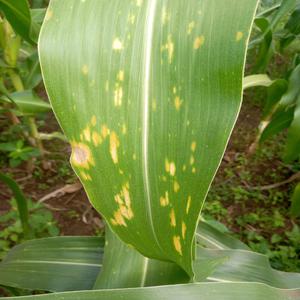

Supplement: S1 File — (ZIP) [file pone.0267650.s001.zip › All data sets/Common rust/IMG_20190523_140602_8.jpg]

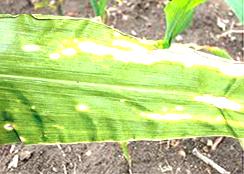

Supplement: S1 File — (ZIP) [file pone.0267650.s001.zip › All data sets/Common rust/brightnessE1.jpg]

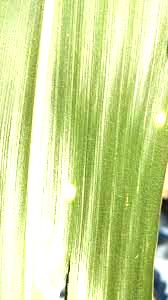

Supplement: S1 File — (ZIP) [file pone.0267650.s001.zip › All data sets/Common rust/brightnessE100.jpg]

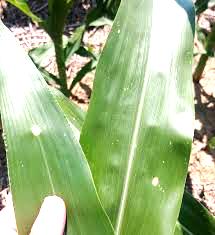

Supplement: S1 File — (ZIP) [file pone.0267650.s001.zip › All data sets/Common rust/brightnessE101.jpg]

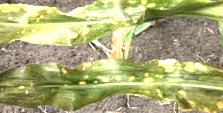

Supplement: S1 File — (ZIP) [file pone.0267650.s001.zip › All data sets/Common rust/brightnessE11.jpg]

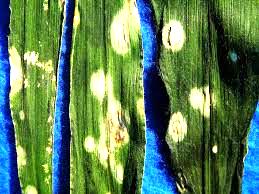

Supplement: S1 File — (ZIP) [file pone.0267650.s001.zip › All data sets/Common rust/brightnessE110.jpg]

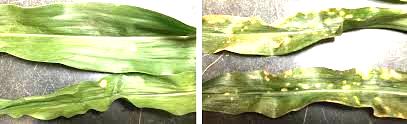

Supplement: S1 File — (ZIP) [file pone.0267650.s001.zip › All data sets/Common rust/brightnessE111.jpg]

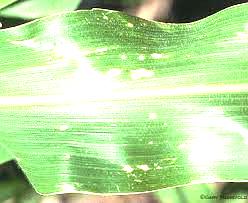

Supplement: S1 File — (ZIP) [file pone.0267650.s001.zip › All data sets/Common rust/brightnessE112.jpg]

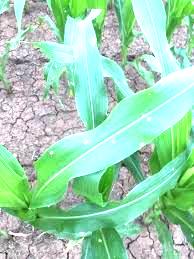

Supplement: S1 File — (ZIP) [file pone.0267650.s001.zip › All data sets/Common rust/brightnessE114.jpg]

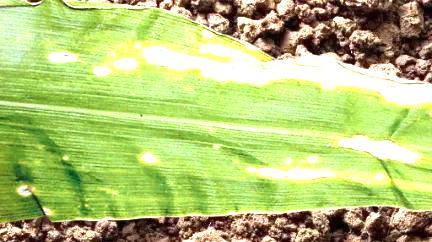

Supplement: S1 File — (ZIP) [file pone.0267650.s001.zip › All data sets/Common rust/brightnessE115.jpg]

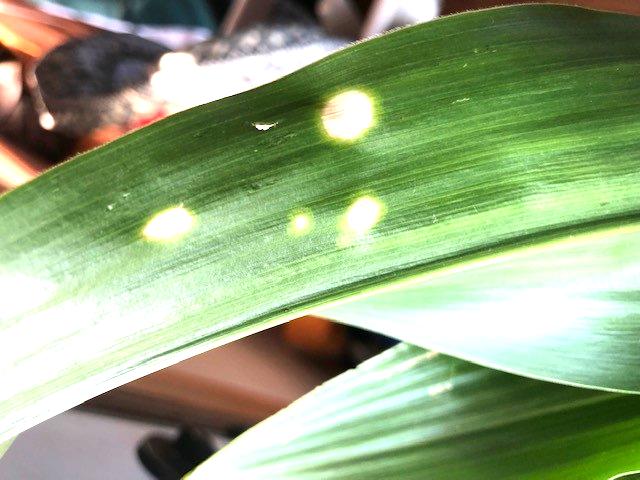

Supplement: S1 File — (ZIP) [file pone.0267650.s001.zip › All data sets/Common rust/brightnessE116.jpg]

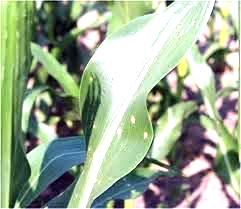

Supplement: S1 File — (ZIP) [file pone.0267650.s001.zip › All data sets/Common rust/brightnessE118.jpg]

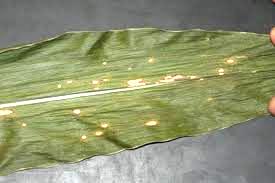

Supplement: S1 File — (ZIP) [file pone.0267650.s001.zip › All data sets/Common rust/brightnessE119.jpg]

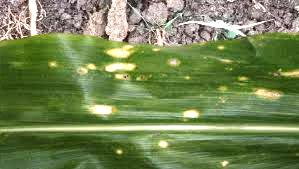

Supplement: S1 File — (ZIP) [file pone.0267650.s001.zip › All data sets/Common rust/brightnessE12.jpg]

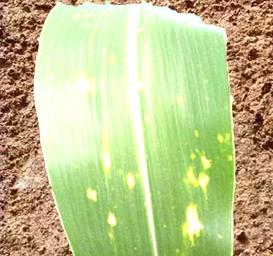

Supplement: S1 File — (ZIP) [file pone.0267650.s001.zip › All data sets/Common rust/brightnessE121.jpg]

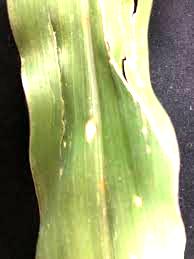

Supplement: S1 File — (ZIP) [file pone.0267650.s001.zip › All data sets/Common rust/brightnessE122.jpg]

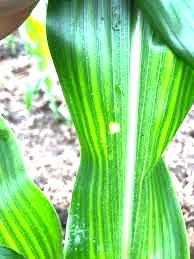

Supplement: S1 File — (ZIP) [file pone.0267650.s001.zip › All data sets/Common rust/brightnessE123.jpg]

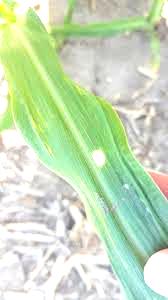

Supplement: S1 File — (ZIP) [file pone.0267650.s001.zip › All data sets/Common rust/brightnessE124.jpg]

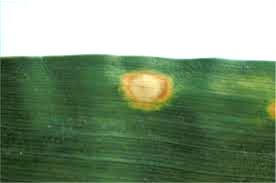

Supplement: S1 File — (ZIP) [file pone.0267650.s001.zip › All data sets/Common rust/brightnessE125.jpg]

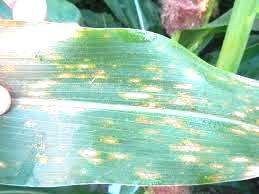

Supplement: S1 File — (ZIP) [file pone.0267650.s001.zip › All data sets/Common rust/brightnessE127.jpg]

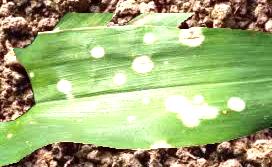

Supplement: S1 File — (ZIP) [file pone.0267650.s001.zip › All data sets/Common rust/brightnessE128.jpg]

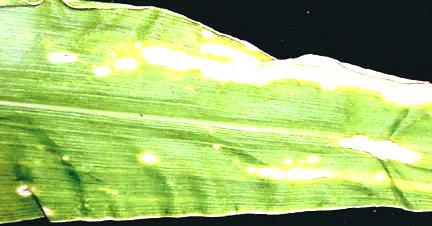

Supplement: S1 File — (ZIP) [file pone.0267650.s001.zip › All data sets/Common rust/brightnessE129.jpg]

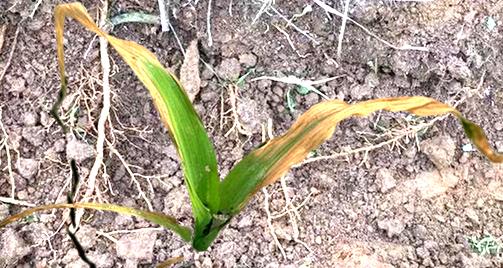

Supplement: S1 File — (ZIP) [file pone.0267650.s001.zip › All data sets/Common rust/brightnessE13.jpg]

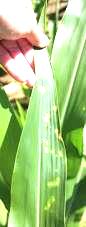

Supplement: S1 File — (ZIP) [file pone.0267650.s001.zip › All data sets/Common rust/brightnessE130.jpg]

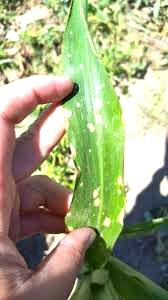

Supplement: S1 File — (ZIP) [file pone.0267650.s001.zip › All data sets/Common rust/brightnessE131.jpg]

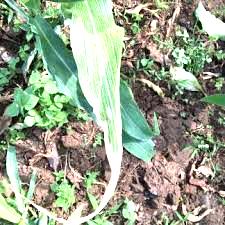

Supplement: S1 File — (ZIP) [file pone.0267650.s001.zip › All data sets/Common rust/brightnessE132.jpg]

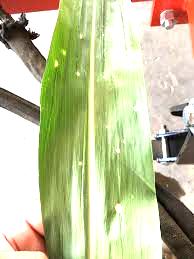

Supplement: S1 File — (ZIP) [file pone.0267650.s001.zip › All data sets/Common rust/brightnessE133.jpg]

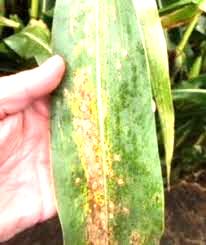

Supplement: S1 File — (ZIP) [file pone.0267650.s001.zip › All data sets/Common rust/brightnessE134.jpg]

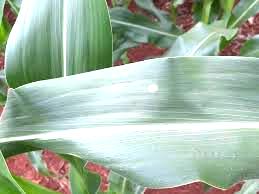

Supplement: S1 File — (ZIP) [file pone.0267650.s001.zip › All data sets/Common rust/brightnessE135.jpg]

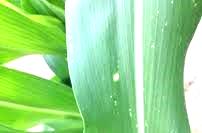

Supplement: S1 File — (ZIP) [file pone.0267650.s001.zip › All data sets/Common rust/brightnessE136.jpg]

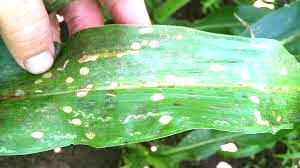

Supplement: S1 File — (ZIP) [file pone.0267650.s001.zip › All data sets/Common rust/brightnessE137.jpg]

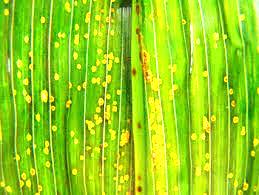

Supplement: S1 File — (ZIP) [file pone.0267650.s001.zip › All data sets/Common rust/brightnessE138.jpg]

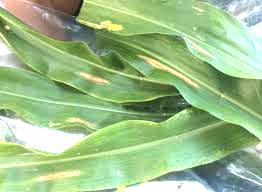

Supplement: S1 File — (ZIP) [file pone.0267650.s001.zip › All data sets/Common rust/brightnessE139.jpg]

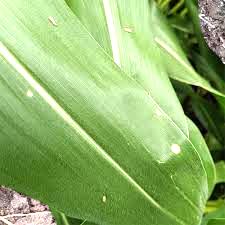

Supplement: S1 File — (ZIP) [file pone.0267650.s001.zip › All data sets/Common rust/brightnessE14.jpg]

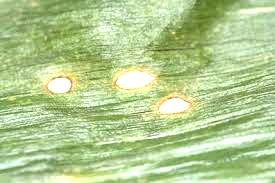

Supplement: S1 File — (ZIP) [file pone.0267650.s001.zip › All data sets/Common rust/brightnessE140.jpg]

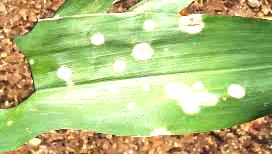

Supplement: S1 File — (ZIP) [file pone.0267650.s001.zip › All data sets/Common rust/brightnessE141.jpg]

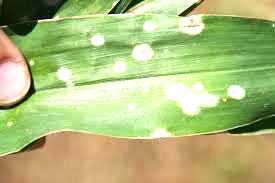

Supplement: S1 File — (ZIP) [file pone.0267650.s001.zip › All data sets/Common rust/brightnessE142.jpg]

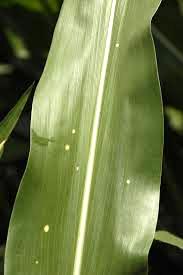

Supplement: S1 File — (ZIP) [file pone.0267650.s001.zip › All data sets/Common rust/brightnessE143.jpg]

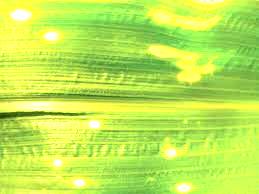

Supplement: S1 File — (ZIP) [file pone.0267650.s001.zip › All data sets/Common rust/brightnessE144.jpg]

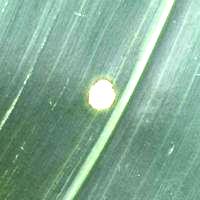

Supplement: S1 File — (ZIP) [file pone.0267650.s001.zip › All data sets/Common rust/brightnessE145.jpg]

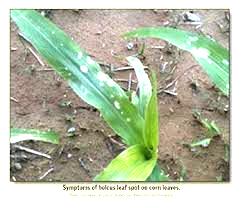

Supplement: S1 File — (ZIP) [file pone.0267650.s001.zip › All data sets/Common rust/brightnessE146.jpg]

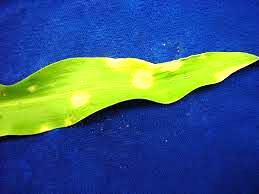

Supplement: S1 File — (ZIP) [file pone.0267650.s001.zip › All data sets/Common rust/brightnessE148.jpg]

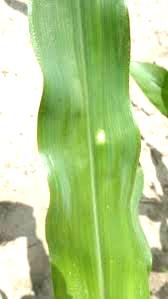

Supplement: S1 File — (ZIP) [file pone.0267650.s001.zip › All data sets/Common rust/brightnessE149.jpg]

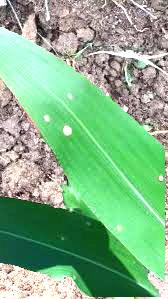

Supplement: S1 File — (ZIP) [file pone.0267650.s001.zip › All data sets/Common rust/brightnessE15.jpg]

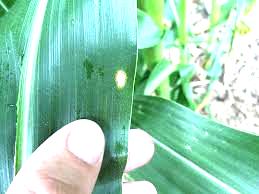

Supplement: S1 File — (ZIP) [file pone.0267650.s001.zip › All data sets/Common rust/brightnessE151.jpg]

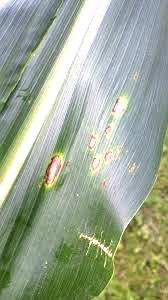

Supplement: S1 File — (ZIP) [file pone.0267650.s001.zip › All data sets/Common rust/brightnessE152.jpg]

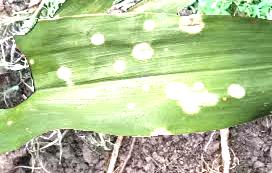

Supplement: S1 File — (ZIP) [file pone.0267650.s001.zip › All data sets/Common rust/brightnessE153.jpg]

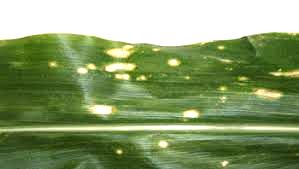

Supplement: S1 File — (ZIP) [file pone.0267650.s001.zip › All data sets/Common rust/brightnessE154.jpg]

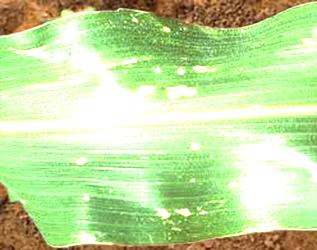

Supplement: S1 File — (ZIP) [file pone.0267650.s001.zip › All data sets/Common rust/brightnessE155.jpg]

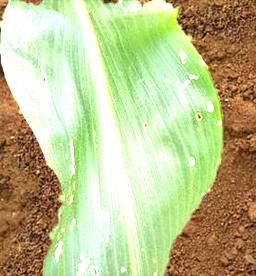

Supplement: S1 File — (ZIP) [file pone.0267650.s001.zip › All data sets/Common rust/brightnessE156.jpg]

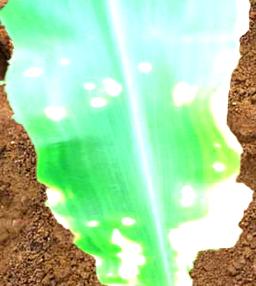

Supplement: S1 File — (ZIP) [file pone.0267650.s001.zip › All data sets/Common rust/brightnessE157.jpg]

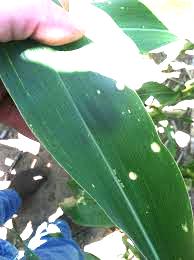

Supplement: S1 File — (ZIP) [file pone.0267650.s001.zip › All data sets/Common rust/brightnessE159.jpg]

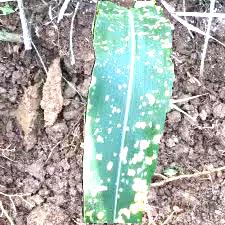

Supplement: S1 File — (ZIP) [file pone.0267650.s001.zip › All data sets/Common rust/brightnessE16.jpg]

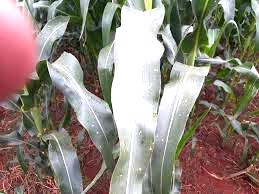

Supplement: S1 File — (ZIP) [file pone.0267650.s001.zip › All data sets/Common rust/brightnessE160.jpg]

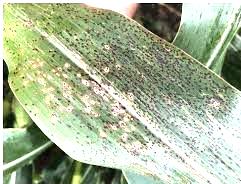

Supplement: S1 File — (ZIP) [file pone.0267650.s001.zip › All data sets/Common rust/brightnessE161.jpg]

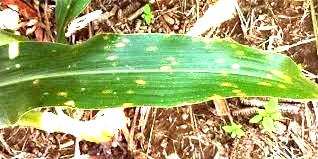

Supplement: S1 File — (ZIP) [file pone.0267650.s001.zip › All data sets/Common rust/brightnessE163.jpg]

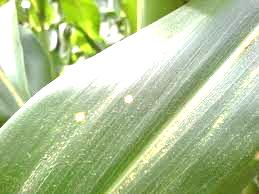

Supplement: S1 File — (ZIP) [file pone.0267650.s001.zip › All data sets/Common rust/brightnessE164.jpg]

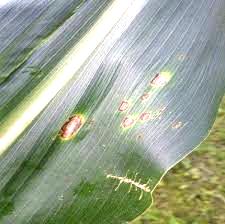

Supplement: S1 File — (ZIP) [file pone.0267650.s001.zip › All data sets/Common rust/brightnessE165.jpg]

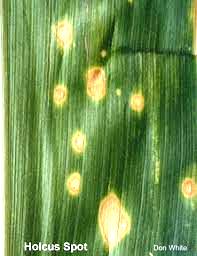

Supplement: S1 File — (ZIP) [file pone.0267650.s001.zip › All data sets/Common rust/brightnessE166.jpg]

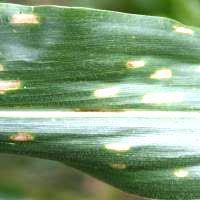

Supplement: S1 File — (ZIP) [file pone.0267650.s001.zip › All data sets/Common rust/brightnessE167.jpg]

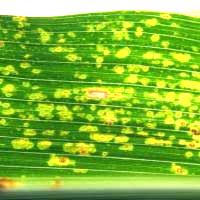

Supplement: S1 File — (ZIP) [file pone.0267650.s001.zip › All data sets/Common rust/brightnessE168.jpg]

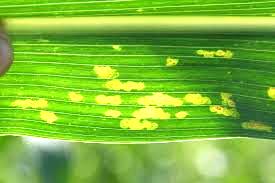

Supplement: S1 File — (ZIP) [file pone.0267650.s001.zip › All data sets/Common rust/brightnessE171.jpg]

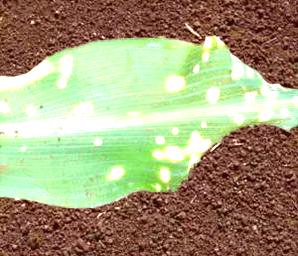

Supplement: S1 File — (ZIP) [file pone.0267650.s001.zip › All data sets/Common rust/brightnessE172.jpg]

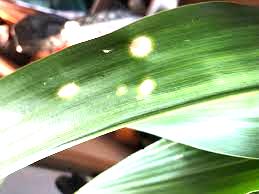

Supplement: S1 File — (ZIP) [file pone.0267650.s001.zip › All data sets/Common rust/brightnessE176.jpg]

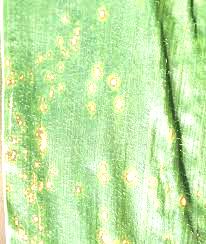

Supplement: S1 File — (ZIP) [file pone.0267650.s001.zip › All data sets/Common rust/brightnessE177.jpg]

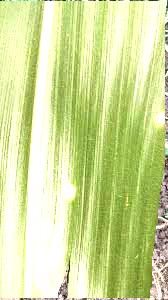

Supplement: S1 File — (ZIP) [file pone.0267650.s001.zip › All data sets/Common rust/brightnessE18.jpg]

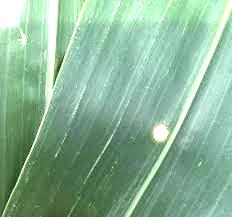

Supplement: S1 File — (ZIP) [file pone.0267650.s001.zip › All data sets/Common rust/brightnessE180.jpg]

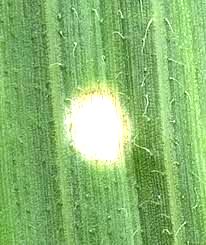

Supplement: S1 File — (ZIP) [file pone.0267650.s001.zip › All data sets/Common rust/brightnessE182.jpg]

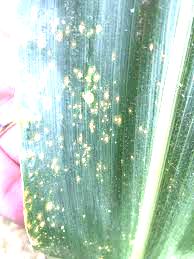

Supplement: S1 File — (ZIP) [file pone.0267650.s001.zip › All data sets/Common rust/brightnessE184.jpg]

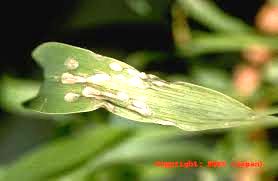

Supplement: S1 File — (ZIP) [file pone.0267650.s001.zip › All data sets/Common rust/brightnessE185.jpg]

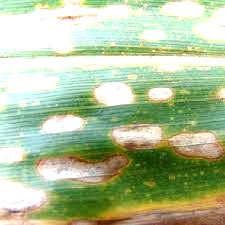

Supplement: S1 File — (ZIP) [file pone.0267650.s001.zip › All data sets/Common rust/brightnessE186.jpg]

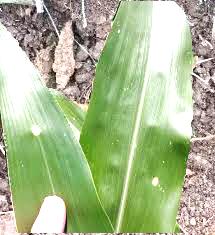

Supplement: S1 File — (ZIP) [file pone.0267650.s001.zip › All data sets/Common rust/brightnessE19.jpg]

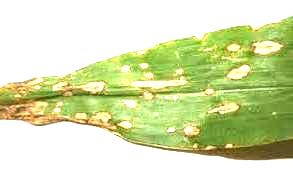

Supplement: S1 File — (ZIP) [file pone.0267650.s001.zip › All data sets/Common rust/brightnessE196.jpg]

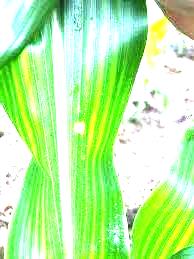

Supplement: S1 File — (ZIP) [file pone.0267650.s001.zip › All data sets/Common rust/brightnessE197.jpg]

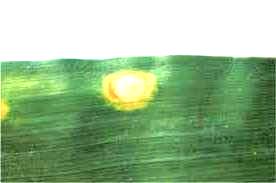

Supplement: S1 File — (ZIP) [file pone.0267650.s001.zip › All data sets/Common rust/brightnessE198.jpg]

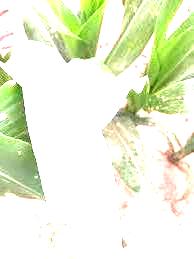

Supplement: S1 File — (ZIP) [file pone.0267650.s001.zip › All data sets/Common rust/brightnessE199.jpg]

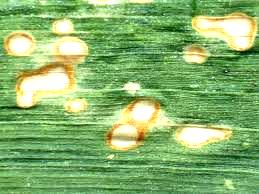

Supplement: S1 File — (ZIP) [file pone.0267650.s001.zip › All data sets/Common rust/brightnessE2.jpg]

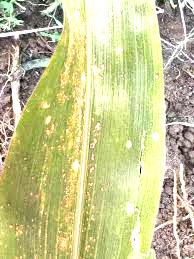

Supplement: S1 File — (ZIP) [file pone.0267650.s001.zip › All data sets/Common rust/brightnessE20.jpg]

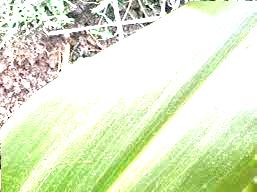

Supplement: S1 File — (ZIP) [file pone.0267650.s001.zip › All data sets/Common rust/brightnessE200.jpg]

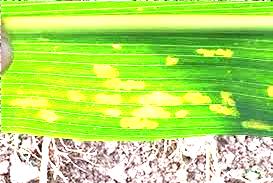

Supplement: S1 File — (ZIP) [file pone.0267650.s001.zip › All data sets/Common rust/brightnessE201.jpg]

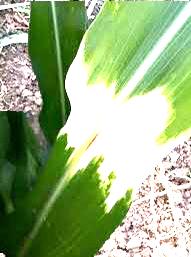

Supplement: S1 File — (ZIP) [file pone.0267650.s001.zip › All data sets/Common rust/brightnessE202.jpg]

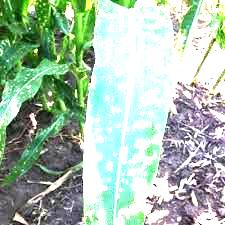

Supplement: S1 File — (ZIP) [file pone.0267650.s001.zip › All data sets/Common rust/brightnessE203.jpg]

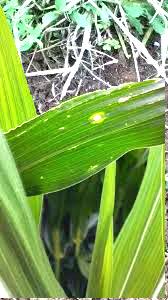

Supplement: S1 File — (ZIP) [file pone.0267650.s001.zip › All data sets/Common rust/brightnessE21.jpg]

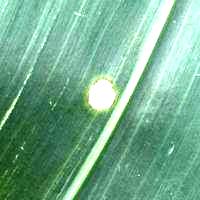

Supplement: S1 File — (ZIP) [file pone.0267650.s001.zip › All data sets/Common rust/brightnessE212.jpg]

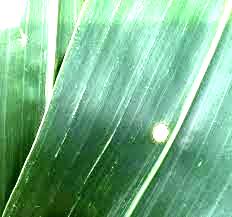

Supplement: S1 File — (ZIP) [file pone.0267650.s001.zip › All data sets/Common rust/brightnessE213.jpg]

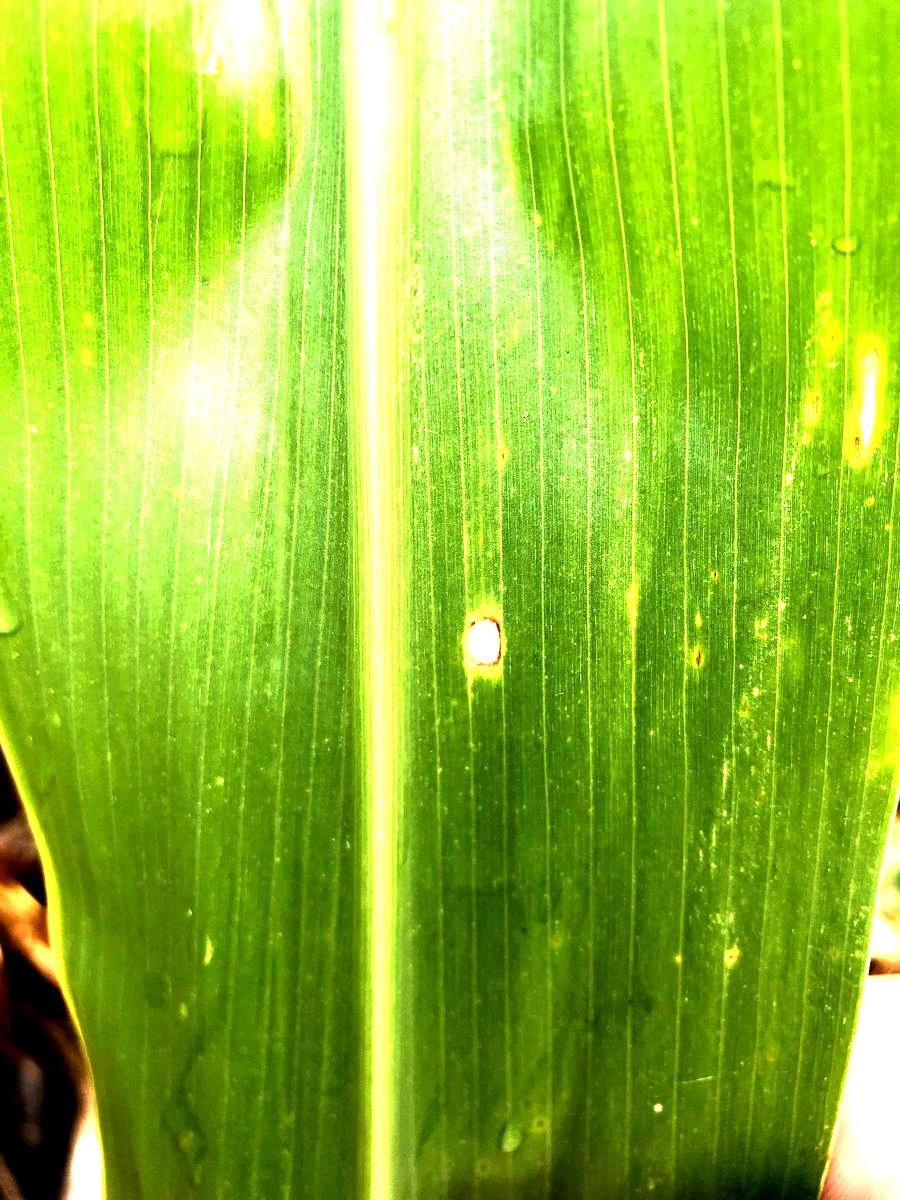

Supplement: S1 File — (ZIP) [file pone.0267650.s001.zip › All data sets/Common rust/brightnessE214.jpg]

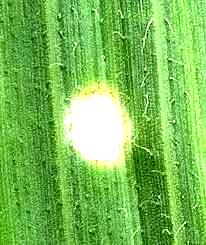

Supplement: S1 File — (ZIP) [file pone.0267650.s001.zip › All data sets/Common rust/brightnessE215.jpg]
