# Supplementary figures and images for: Maize leaf disease identification based on WG-MARNet (part 1 of 2)
Source: PLoS One. 2022 Apr 28;17(4):e0267650. doi: 10.1371/journal.pone.0267650 (PMC9050012; doi:10.1371/journal.pone.0267650)

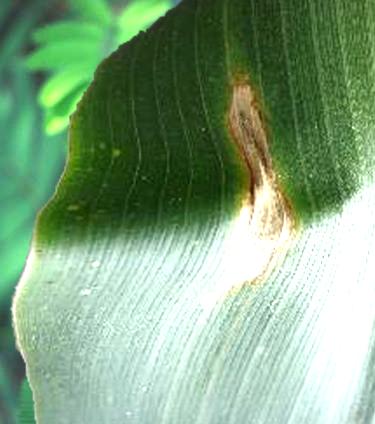

Supplement: S1 File — (ZIP) [file pone.0267650.s001.zip › All data sets/Anthracnose leaf blight/brightnessE242.jpg]

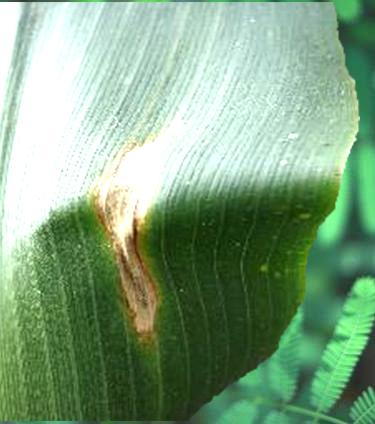

Supplement: S1 File — (ZIP) [file pone.0267650.s001.zip › All data sets/Anthracnose leaf blight/brightnessE243.jpg]

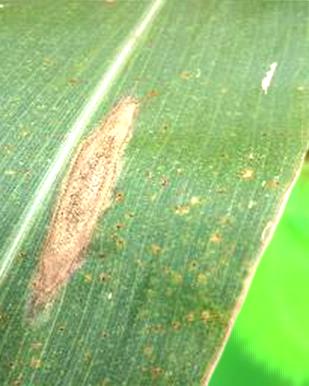

Supplement: S1 File — (ZIP) [file pone.0267650.s001.zip › All data sets/Anthracnose leaf blight/brightnessE244.jpg]

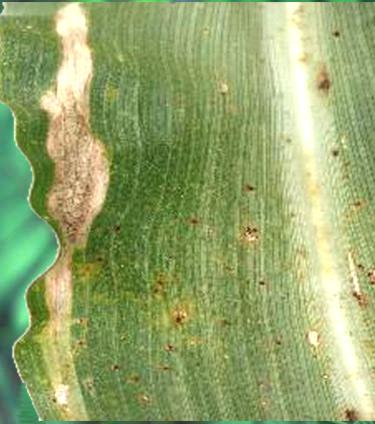

Supplement: S1 File — (ZIP) [file pone.0267650.s001.zip › All data sets/Anthracnose leaf blight/brightnessE245.jpg]

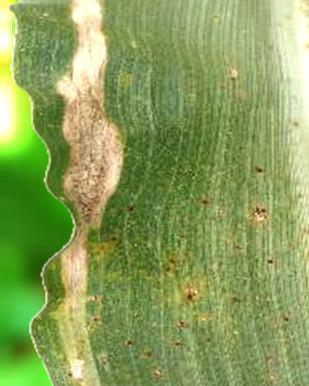

Supplement: S1 File — (ZIP) [file pone.0267650.s001.zip › All data sets/Anthracnose leaf blight/brightnessE246.jpg]

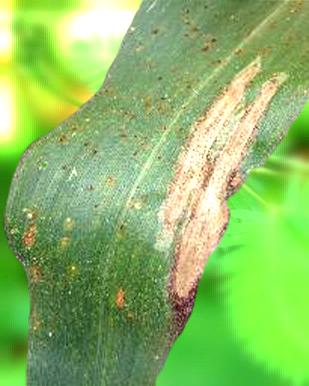

Supplement: S1 File — (ZIP) [file pone.0267650.s001.zip › All data sets/Anthracnose leaf blight/brightnessE247.jpg]

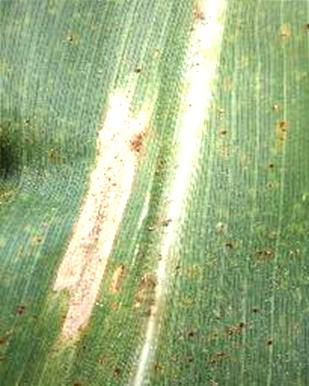

Supplement: S1 File — (ZIP) [file pone.0267650.s001.zip › All data sets/Anthracnose leaf blight/brightnessE249.jpg]

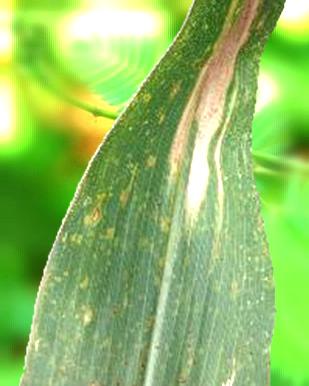

Supplement: S1 File — (ZIP) [file pone.0267650.s001.zip › All data sets/Anthracnose leaf blight/brightnessE250.jpg]

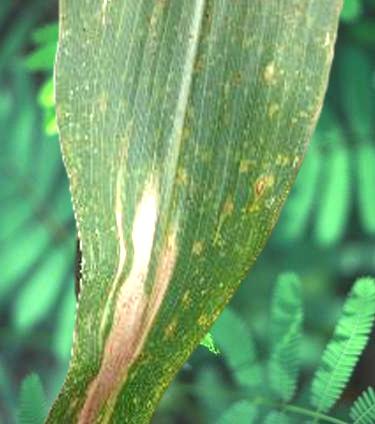

Supplement: S1 File — (ZIP) [file pone.0267650.s001.zip › All data sets/Anthracnose leaf blight/brightnessE251.jpg]

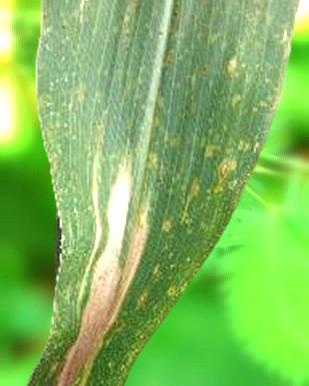

Supplement: S1 File — (ZIP) [file pone.0267650.s001.zip › All data sets/Anthracnose leaf blight/brightnessE252.jpg]

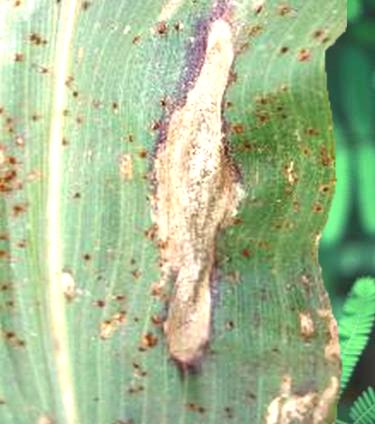

Supplement: S1 File — (ZIP) [file pone.0267650.s001.zip › All data sets/Anthracnose leaf blight/brightnessE253.jpg]

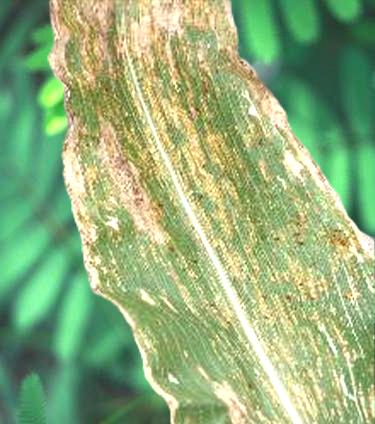

Supplement: S1 File — (ZIP) [file pone.0267650.s001.zip › All data sets/Anthracnose leaf blight/brightnessE254.jpg]

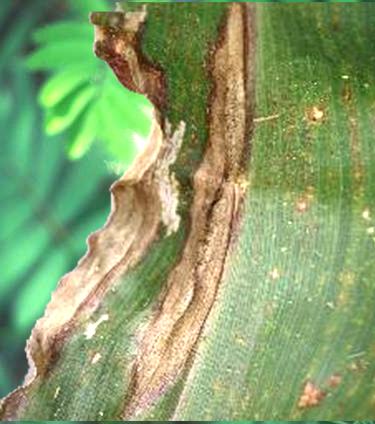

Supplement: S1 File — (ZIP) [file pone.0267650.s001.zip › All data sets/Anthracnose leaf blight/brightnessE255.jpg]

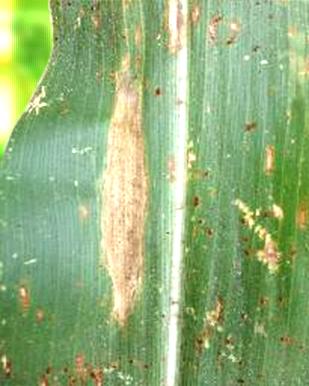

Supplement: S1 File — (ZIP) [file pone.0267650.s001.zip › All data sets/Anthracnose leaf blight/brightnessE256.jpg]

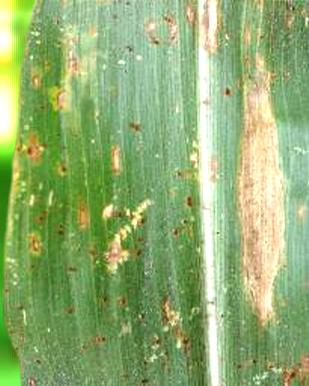

Supplement: S1 File — (ZIP) [file pone.0267650.s001.zip › All data sets/Anthracnose leaf blight/brightnessE257.jpg]

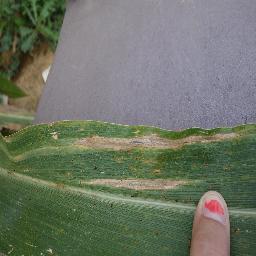

Supplement: S1 File — (ZIP) [file pone.0267650.s001.zip › All data sets/Anthracnose leaf blight/d08114ac-a98f-41eb-82f9-fdfa91446ed7___RS_NLB4062.JPG]

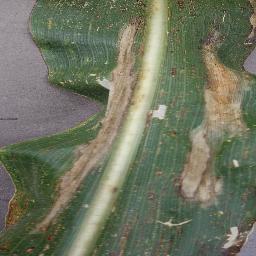

Supplement: S1 File — (ZIP) [file pone.0267650.s001.zip › All data sets/Anthracnose leaf blight/d1265756-90d5-4126-943b-4bff5b1e8277___RS_NLB3904.JPG]

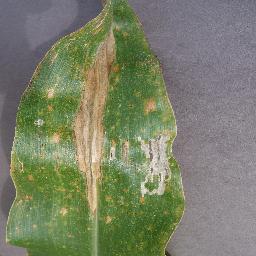

Supplement: S1 File — (ZIP) [file pone.0267650.s001.zip › All data sets/Anthracnose leaf blight/d312ede5-f6d0-4446-8b4d-d0ae6e13864f___RS_NLB4169.JPG]

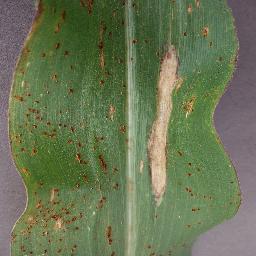

Supplement: S1 File — (ZIP) [file pone.0267650.s001.zip › All data sets/Anthracnose leaf blight/d330ac59-dc5f-49d4-9529-a4ebbc34d46b___RS_NLB3531.JPG]

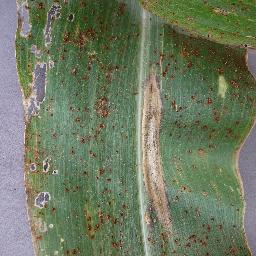

Supplement: S1 File — (ZIP) [file pone.0267650.s001.zip › All data sets/Anthracnose leaf blight/d334d37f-a75c-47d9-ad48-3d37c32ea3c9___RS_NLB3582.JPG]

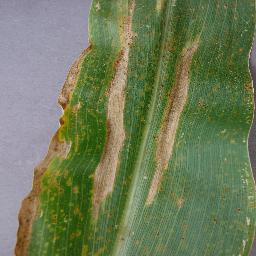

Supplement: S1 File — (ZIP) [file pone.0267650.s001.zip › All data sets/Anthracnose leaf blight/d9987503-f4fb-4ed8-a766-dcc4bc973fb0___RS_NLB4070.JPG]

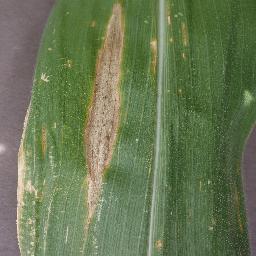

Supplement: S1 File — (ZIP) [file pone.0267650.s001.zip › All data sets/Anthracnose leaf blight/da24c7cf-a119-46e9-b34a-049d21fe610c___RS_NLB0831.JPG]

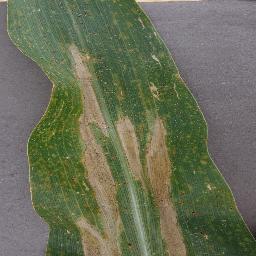

Supplement: S1 File — (ZIP) [file pone.0267650.s001.zip › All data sets/Anthracnose leaf blight/da98944f-f66b-4734-9d09-58dbb072eb85___RS_NLB4033.JPG]

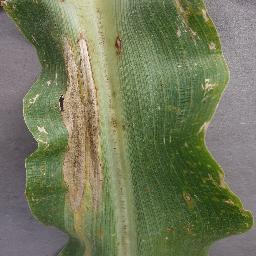

Supplement: S1 File — (ZIP) [file pone.0267650.s001.zip › All data sets/Anthracnose leaf blight/da9a467c-be7b-4164-81a2-74b82f268bb6___RS_NLB4122.JPG]

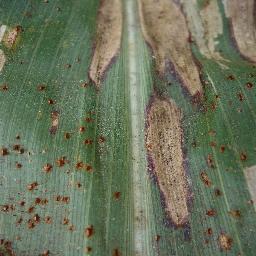

Supplement: S1 File — (ZIP) [file pone.0267650.s001.zip › All data sets/Anthracnose leaf blight/dac28004-9243-45ab-a3ff-03edff04e8d1___RS_NLB3613.JPG]

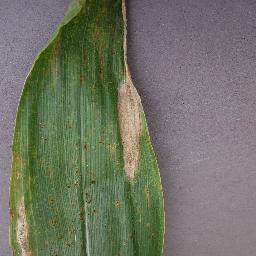

Supplement: S1 File — (ZIP) [file pone.0267650.s001.zip › All data sets/Anthracnose leaf blight/daf1c801-8722-44d6-9f3f-be0250d952e6___RS_NLB3941.JPG]

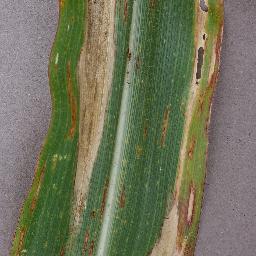

Supplement: S1 File — (ZIP) [file pone.0267650.s001.zip › All data sets/Anthracnose leaf blight/dbc0dc71-c477-4d79-9c13-479bbbe783c8___RS_NLB3931copy.jpg]

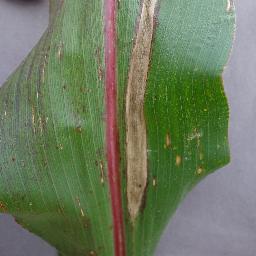

Supplement: S1 File — (ZIP) [file pone.0267650.s001.zip › All data sets/Anthracnose leaf blight/dc5013d6-de04-4079-93f1-320d6b765cd3___RS_NLB0822copy.jpg]

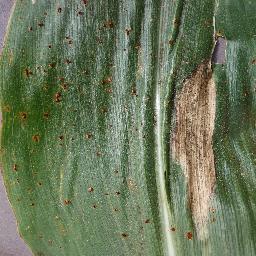

Supplement: S1 File — (ZIP) [file pone.0267650.s001.zip › All data sets/Anthracnose leaf blight/dca191ee-4869-454f-a67e-6af114398edf___RS_NLB3647.JPG]

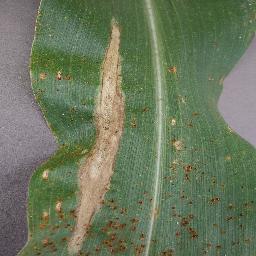

Supplement: S1 File — (ZIP) [file pone.0267650.s001.zip › All data sets/Anthracnose leaf blight/dda73df7-96ed-453c-8d53-21549a980bc7___RS_NLB4182.JPG]

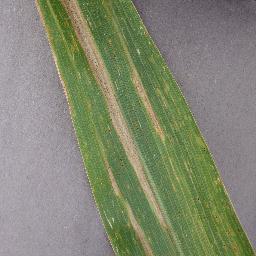

Supplement: S1 File — (ZIP) [file pone.0267650.s001.zip › All data sets/Anthracnose leaf blight/ddd3bb1b-ef88-4426-a7bb-2b61422df6e2___RS_NLB4057copy3.jpg]

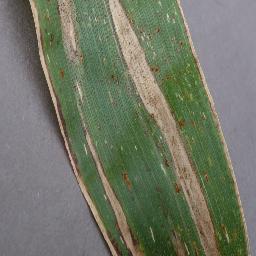

Supplement: S1 File — (ZIP) [file pone.0267650.s001.zip › All data sets/Anthracnose leaf blight/de023021-3b7a-4b88-bb83-7bcb09f80b6a___RS_NLB4666copy.jpg]

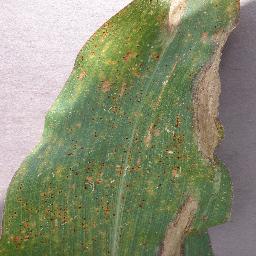

Supplement: S1 File — (ZIP) [file pone.0267650.s001.zip › All data sets/Anthracnose leaf blight/de06fdbb-04cf-4e7b-9e1d-6162f11b3dea___RS_NLB3481.JPG]

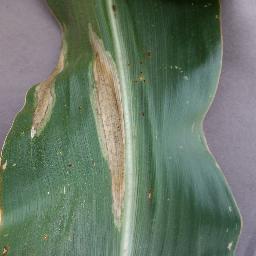

Supplement: S1 File — (ZIP) [file pone.0267650.s001.zip › All data sets/Anthracnose leaf blight/def2064a-8470-447e-bfb2-f76ecb3006cd___RS_NLB0789.JPG]

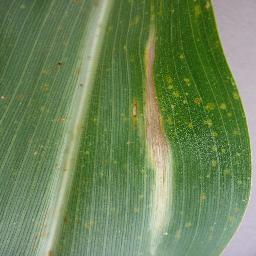

Supplement: S1 File — (ZIP) [file pone.0267650.s001.zip › All data sets/Anthracnose leaf blight/e0246bcb-949d-4ea8-b517-f6dfb7e7526d___RS_NLB4107.JPG]

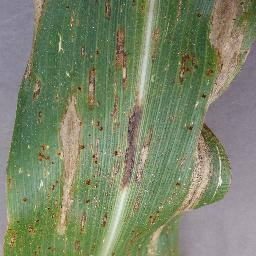

Supplement: S1 File — (ZIP) [file pone.0267650.s001.zip › All data sets/Anthracnose leaf blight/e0b458e7-cea5-4d65-9b14-2aa51ba38438___RS_NLB3857.JPG]

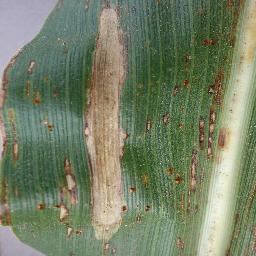

Supplement: S1 File — (ZIP) [file pone.0267650.s001.zip › All data sets/Anthracnose leaf blight/e11c9b3e-d9bc-48b7-8d05-c1d99ba2da52___RS_NLB3851.JPG]

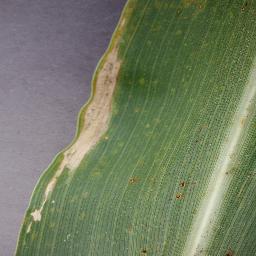

Supplement: S1 File — (ZIP) [file pone.0267650.s001.zip › All data sets/Anthracnose leaf blight/e15e053c-4b0a-48a3-a622-c2395bd93b64___RS_NLB4008.JPG]

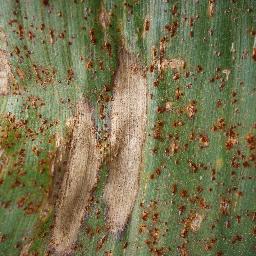

Supplement: S1 File — (ZIP) [file pone.0267650.s001.zip › All data sets/Anthracnose leaf blight/e22976d4-ff48-4672-ad70-e17d50b65b1c___RS_NLB3688.JPG]

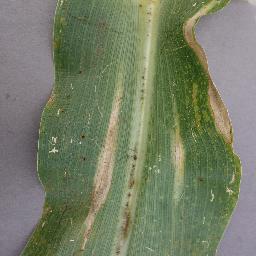

Supplement: S1 File — (ZIP) [file pone.0267650.s001.zip › All data sets/Anthracnose leaf blight/e2456029-abe6-4789-a1da-9f1a884c1ab6___RS_NLB4028.JPG]

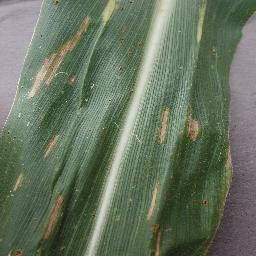

Supplement: S1 File — (ZIP) [file pone.0267650.s001.zip › All data sets/Anthracnose leaf blight/e2faa2e3-433a-4ee3-9b64-8de4ad2947e1___RS_NLB3795.JPG]

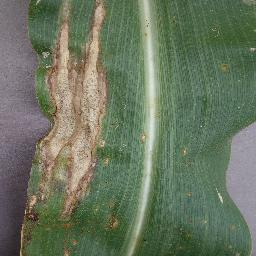

Supplement: S1 File — (ZIP) [file pone.0267650.s001.zip › All data sets/Anthracnose leaf blight/e2fc31a8-6b0e-4c15-9fba-2024d2295827___RS_NLB4229.JPG]

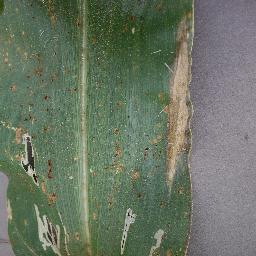

Supplement: S1 File — (ZIP) [file pone.0267650.s001.zip › All data sets/Anthracnose leaf blight/e379b939-5325-400c-97ea-393159297fc0___RS_NLB4184.JPG]

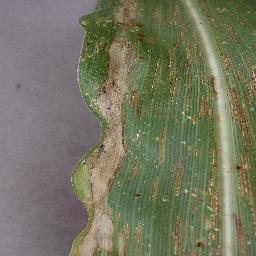

Supplement: S1 File — (ZIP) [file pone.0267650.s001.zip › All data sets/Anthracnose leaf blight/e472dfcf-e2c6-4e5b-9d93-b31921cb5f07___RS_NLB3974.JPG]

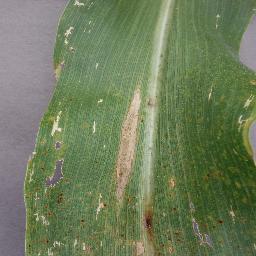

Supplement: S1 File — (ZIP) [file pone.0267650.s001.zip › All data sets/Anthracnose leaf blight/e59d2407-e225-47d5-96a7-6cdcc3868499___RS_NLB4100.JPG]

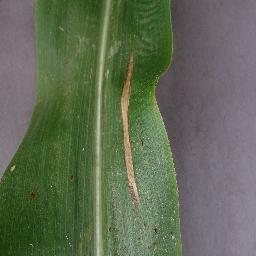

Supplement: S1 File — (ZIP) [file pone.0267650.s001.zip › All data sets/Anthracnose leaf blight/e60c841c-2a10-4a27-a2e9-2604fb9ead83___RS_NLB4192.JPG]

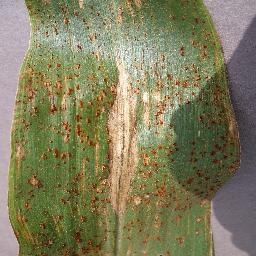

Supplement: S1 File — (ZIP) [file pone.0267650.s001.zip › All data sets/Anthracnose leaf blight/e617de29-f6fb-4c0b-9995-49127fb4573c___RS_NLB3601.JPG]

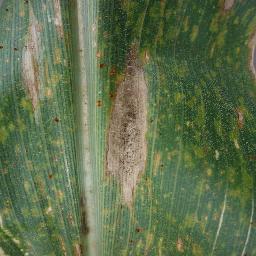

Supplement: S1 File — (ZIP) [file pone.0267650.s001.zip › All data sets/Anthracnose leaf blight/e6f10c6e-9cd7-4ad1-98d8-0fb8038e4c1a___RS_NLB0820.JPG]

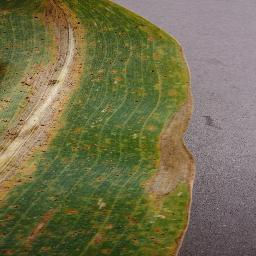

Supplement: S1 File — (ZIP) [file pone.0267650.s001.zip › All data sets/Anthracnose leaf blight/e76e03e7-ee83-4c2e-9780-188e6fde75a1___RS_NLB4007.JPG]

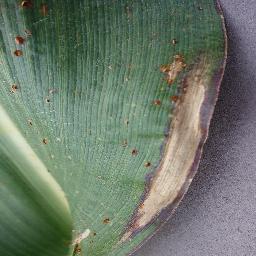

Supplement: S1 File — (ZIP) [file pone.0267650.s001.zip › All data sets/Anthracnose leaf blight/e863323a-5d81-42c8-8503-dbdb00452e59___RS_NLB3554.JPG]

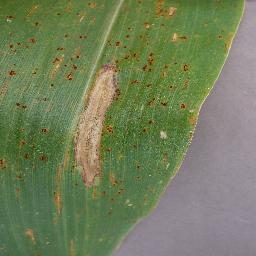

Supplement: S1 File — (ZIP) [file pone.0267650.s001.zip › All data sets/Anthracnose leaf blight/e9b6bee3-c097-4823-b057-c7ed167f072c___RS_NLB4151.JPG]

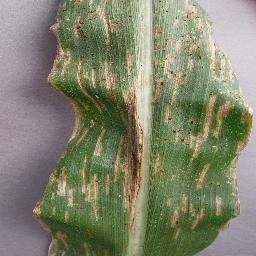

Supplement: S1 File — (ZIP) [file pone.0267650.s001.zip › All data sets/Anthracnose leaf blight/ea41c5a7-8436-4ee5-b528-4c0f5b656e82___RS_NLB3786.JPG]

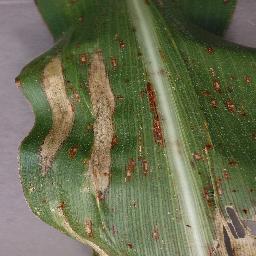

Supplement: S1 File — (ZIP) [file pone.0267650.s001.zip › All data sets/Anthracnose leaf blight/ea5341a5-d8b9-4e96-a3c8-7785e02d0341___RS_NLB3816.JPG]

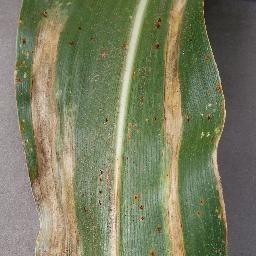

Supplement: S1 File — (ZIP) [file pone.0267650.s001.zip › All data sets/Anthracnose leaf blight/ea9ba5b2-e8e7-48c8-ac16-d594a9e5cc51___RS_NLB4443copy2.jpg]

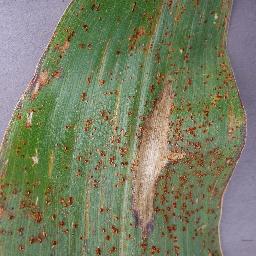

Supplement: S1 File — (ZIP) [file pone.0267650.s001.zip › All data sets/Anthracnose leaf blight/eac45516-3f72-4dac-bf0f-566a663c1409___RS_NLB3580.JPG]

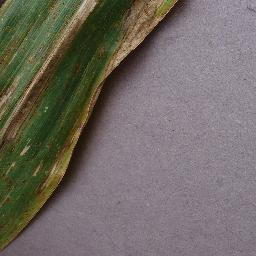

Supplement: S1 File — (ZIP) [file pone.0267650.s001.zip › All data sets/Anthracnose leaf blight/eaf93f13-37be-489a-9405-b7602bf59f8a___RS_NLB3963copy.jpg]

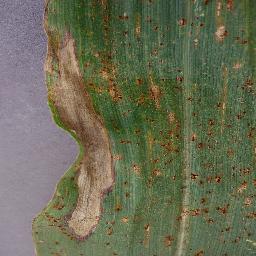

Supplement: S1 File — (ZIP) [file pone.0267650.s001.zip › All data sets/Anthracnose leaf blight/ebb837be-867e-417a-8a0a-04fd3f0570ba___RS_NLB4087.JPG]

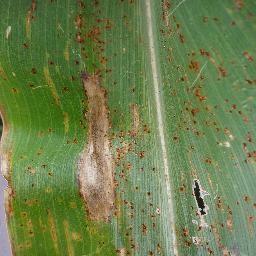

Supplement: S1 File — (ZIP) [file pone.0267650.s001.zip › All data sets/Anthracnose leaf blight/ebcfe20f-f00e-49ad-a470-4b4cc40b815c___RS_NLB3742.JPG]

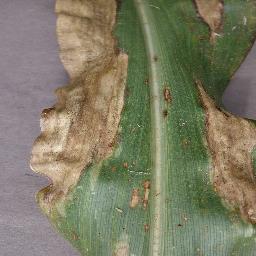

Supplement: S1 File — (ZIP) [file pone.0267650.s001.zip › All data sets/Anthracnose leaf blight/ec769dd1-532c-4f81-b677-6b7b4d0d7606___RS_NLB4140.JPG]

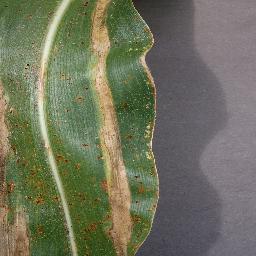

Supplement: S1 File — (ZIP) [file pone.0267650.s001.zip › All data sets/Anthracnose leaf blight/ed02da2f-8b98-49b1-a65d-ff7e47d61331___RS_NLB4443.JPG]

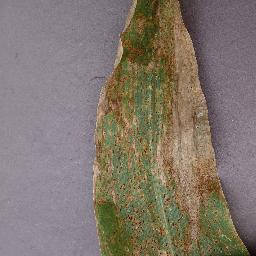

Supplement: S1 File — (ZIP) [file pone.0267650.s001.zip › All data sets/Anthracnose leaf blight/ed1e71f3-0007-458d-bdd0-eaceeee65707___RS_NLB4224.JPG]

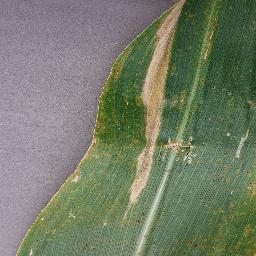

Supplement: S1 File — (ZIP) [file pone.0267650.s001.zip › All data sets/Anthracnose leaf blight/ed700cb4-fa84-4c56-bcf4-f2c21ce53e12___RS_NLB4040.JPG]

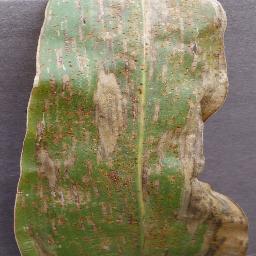

Supplement: S1 File — (ZIP) [file pone.0267650.s001.zip › All data sets/Anthracnose leaf blight/ed711fe2-468f-4c9b-b2fc-32761db16c94___RS_NLB3953.JPG]

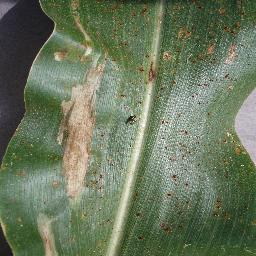

Supplement: S1 File — (ZIP) [file pone.0267650.s001.zip › All data sets/Anthracnose leaf blight/ed87978b-71f0-4ab8-b1d3-e7038c0dfa16___RS_NLB3494.JPG]

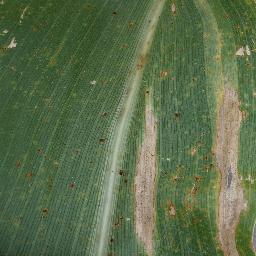

Supplement: S1 File — (ZIP) [file pone.0267650.s001.zip › All data sets/Anthracnose leaf blight/ef51615e-208a-4af4-a392-29abe4c5d008___RS_NLB4062copy2.jpg]

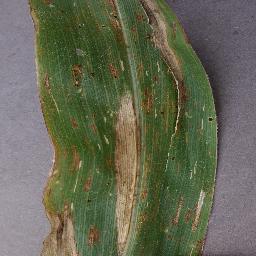

Supplement: S1 File — (ZIP) [file pone.0267650.s001.zip › All data sets/Anthracnose leaf blight/ef5c7a36-4a79-419f-94e9-ad1120a68dad___RS_NLB3932copy.jpg]

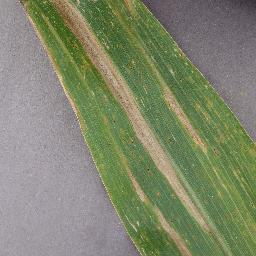

Supplement: S1 File — (ZIP) [file pone.0267650.s001.zip › All data sets/Anthracnose leaf blight/efb9c96a-22d4-4217-bf2b-7c0f5d97d277___RS_NLB4057copy2.jpg]

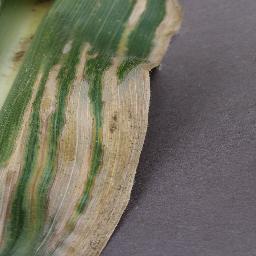

Supplement: S1 File — (ZIP) [file pone.0267650.s001.zip › All data sets/Anthracnose leaf blight/f0b49686-1a0f-4c01-9b60-e816c513215f___RS_NLB4506copy.jpg]

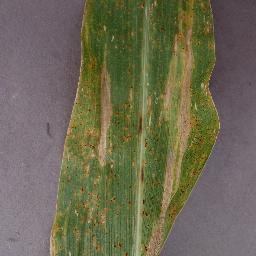

Supplement: S1 File — (ZIP) [file pone.0267650.s001.zip › All data sets/Anthracnose leaf blight/f0edc304-8a96-4b71-bf5b-2a379967ba27___RS_NLB4018.JPG]

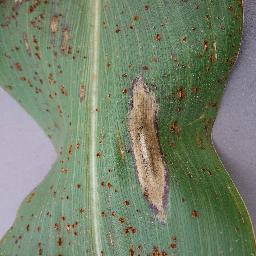

Supplement: S1 File — (ZIP) [file pone.0267650.s001.zip › All data sets/Anthracnose leaf blight/f13d43cb-e365-4df6-aac8-746e4d6d7dab___RS_NLB3528.JPG]

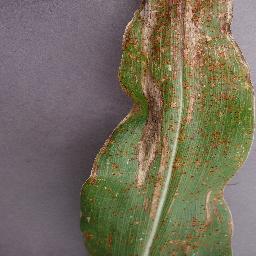

Supplement: S1 File — (ZIP) [file pone.0267650.s001.zip › All data sets/Anthracnose leaf blight/f2486598-1ee4-4fde-9d9a-5684f2cbdae3___RS_NLB3784.JPG]

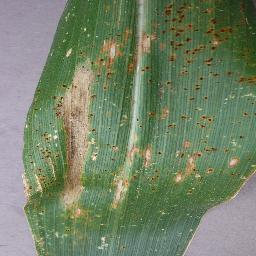

Supplement: S1 File — (ZIP) [file pone.0267650.s001.zip › All data sets/Anthracnose leaf blight/f25476a1-6701-4519-a20e-075643e5359e___RS_NLB0815.JPG]

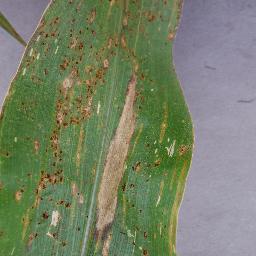

Supplement: S1 File — (ZIP) [file pone.0267650.s001.zip › All data sets/Anthracnose leaf blight/f283c7ad-19cf-4e5f-a2cb-d1123610b077___RS_NLB3573.JPG]

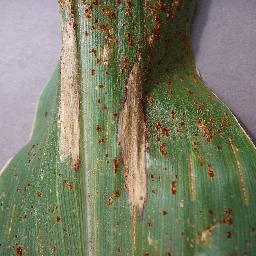

Supplement: S1 File — (ZIP) [file pone.0267650.s001.zip › All data sets/Anthracnose leaf blight/f291f62a-d6f6-4f1e-92af-d6177b154263___RS_NLB3662.JPG]

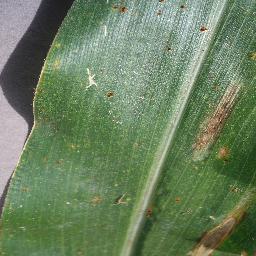

Supplement: S1 File — (ZIP) [file pone.0267650.s001.zip › All data sets/Anthracnose leaf blight/f3141371-5c55-4cf4-988a-6c322de0c6ec___RS_NLB3486copy2.jpg]

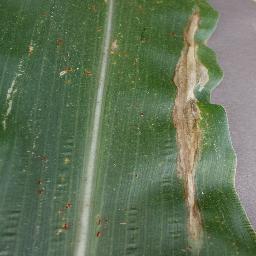

Supplement: S1 File — (ZIP) [file pone.0267650.s001.zip › All data sets/Anthracnose leaf blight/f339c8f8-0de3-427f-86ce-2d3258794ad2___RS_NLB4176.JPG]

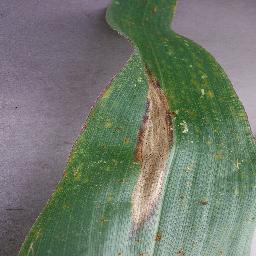

Supplement: S1 File — (ZIP) [file pone.0267650.s001.zip › All data sets/Anthracnose leaf blight/f38dbeb3-7140-4470-911f-7545b495776f___RS_NLB3473.JPG]

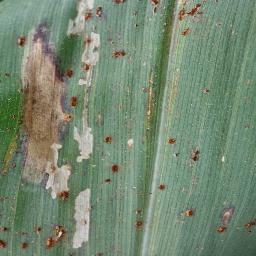

Supplement: S1 File — (ZIP) [file pone.0267650.s001.zip › All data sets/Anthracnose leaf blight/f3a574c8-043a-4804-bdba-addb50450720___RS_NLB3553copy.jpg]

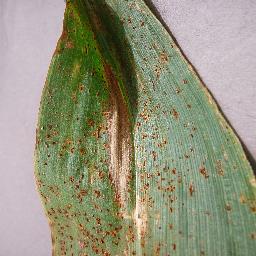

Supplement: S1 File — (ZIP) [file pone.0267650.s001.zip › All data sets/Anthracnose leaf blight/f437c2b5-6299-454b-bb73-b09ebb30cbb3___RS_NLB3664.JPG]

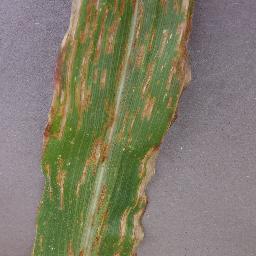

Supplement: S1 File — (ZIP) [file pone.0267650.s001.zip › All data sets/Anthracnose leaf blight/f51cd705-475e-4ced-877a-53422d0d2854___RS_NLB3808.JPG]

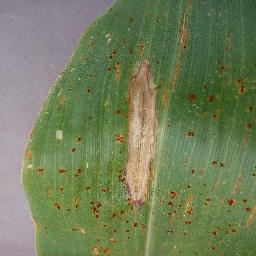

Supplement: S1 File — (ZIP) [file pone.0267650.s001.zip › All data sets/Anthracnose leaf blight/f52ec293-3749-4e11-bcab-2935dcd6eb6d___RS_NLB4150.JPG]

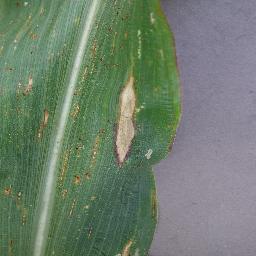

Supplement: S1 File — (ZIP) [file pone.0267650.s001.zip › All data sets/Anthracnose leaf blight/f546ba30-4b0b-4906-a527-bb4834df87cd___RS_NLB3558.JPG]

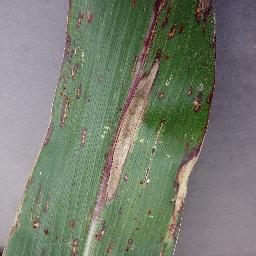

Supplement: S1 File — (ZIP) [file pone.0267650.s001.zip › All data sets/Anthracnose leaf blight/f553d7cf-9c9f-46bf-b99a-8e3505afd96d___RS_NLB3873.JPG]

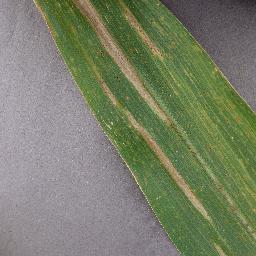

Supplement: S1 File — (ZIP) [file pone.0267650.s001.zip › All data sets/Anthracnose leaf blight/f5773153-728e-4631-8cc8-1bc06412df89___RS_NLB4056.JPG]

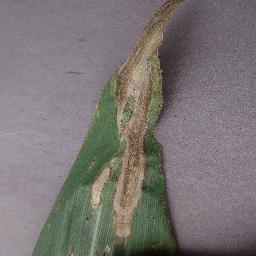

Supplement: S1 File — (ZIP) [file pone.0267650.s001.zip › All data sets/Anthracnose leaf blight/f586f423-9d98-4050-8c90-0b6a3a5e89f8___RS_NLB3862.JPG]

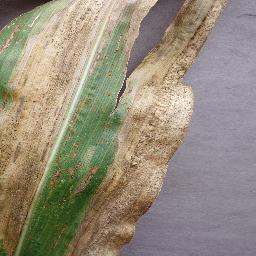

Supplement: S1 File — (ZIP) [file pone.0267650.s001.zip › All data sets/Anthracnose leaf blight/f69b1b10-dd7f-44c0-b084-1959316e4f2a___RS_NLB3955copy2.jpg]

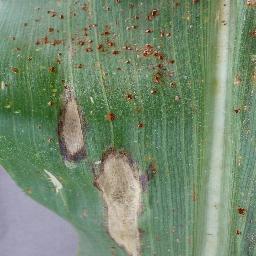

Supplement: S1 File — (ZIP) [file pone.0267650.s001.zip › All data sets/Anthracnose leaf blight/f6b47d16-cfbc-4786-bc6d-78a2bc037a43___RS_NLB3545.JPG]

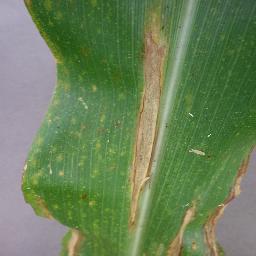

Supplement: S1 File — (ZIP) [file pone.0267650.s001.zip › All data sets/Anthracnose leaf blight/f7cf0608-2278-4a38-a37e-1356bd359651___RS_NLB3490.JPG]

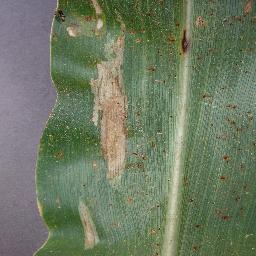

Supplement: S1 File — (ZIP) [file pone.0267650.s001.zip › All data sets/Anthracnose leaf blight/f81a1119-4ccb-46b6-aff7-29998828be59___RS_NLB4187.JPG]

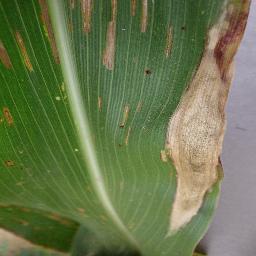

Supplement: S1 File — (ZIP) [file pone.0267650.s001.zip › All data sets/Anthracnose leaf blight/f9276b30-6706-47b2-9396-3799808cc29a___RS_NLB4262.JPG]

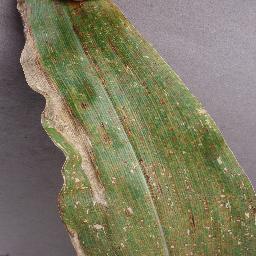

Supplement: S1 File — (ZIP) [file pone.0267650.s001.zip › All data sets/Anthracnose leaf blight/f9e08c76-e2fe-4498-9f90-0b47ffac0e62___RS_NLB3820.JPG]

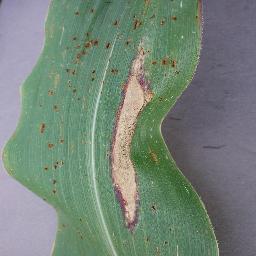

Supplement: S1 File — (ZIP) [file pone.0267650.s001.zip › All data sets/Anthracnose leaf blight/fa30c8e9-b74d-49a2-8611-c9b9098e21c3___RS_NLB3507.JPG]

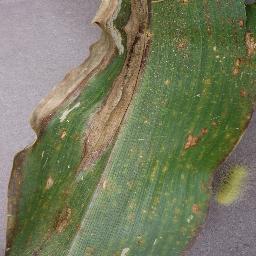

Supplement: S1 File — (ZIP) [file pone.0267650.s001.zip › All data sets/Anthracnose leaf blight/fae01c0a-db82-4189-9193-bdad09ede531___RS_NLB4165copy.jpg]

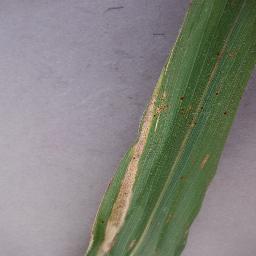

Supplement: S1 File — (ZIP) [file pone.0267650.s001.zip › All data sets/Anthracnose leaf blight/fb1e8075-1546-4c0a-bef1-e18642415358___RS_NLB3966.JPG]

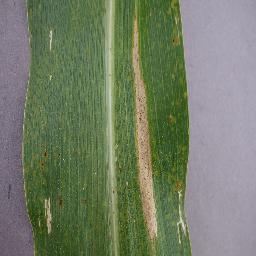

Supplement: S1 File — (ZIP) [file pone.0267650.s001.zip › All data sets/Anthracnose leaf blight/fb83c22d-b379-48c7-bccc-aebdfd19eda0___RS_NLB4252.JPG]

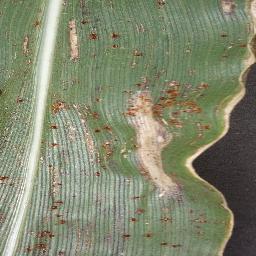

Supplement: S1 File — (ZIP) [file pone.0267650.s001.zip › All data sets/Anthracnose leaf blight/fc19258c-634e-4cc0-b6cf-0144c550ed80___RS_NLB3834copy.jpg]

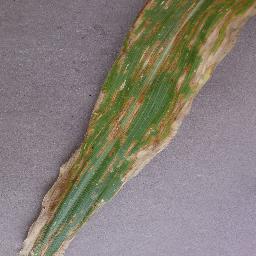

Supplement: S1 File — (ZIP) [file pone.0267650.s001.zip › All data sets/Anthracnose leaf blight/fc6b2569-68e4-49cc-bd4b-c27e037bd3c9___RS_NLB3806.JPG]

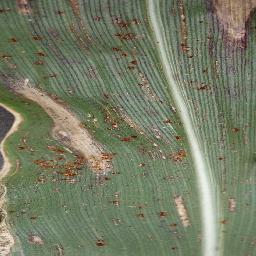

Supplement: S1 File — (ZIP) [file pone.0267650.s001.zip › All data sets/Anthracnose leaf blight/fc84ca09-d6c7-493e-957b-c46b2055b702___RS_NLB3836copy2.jpg]

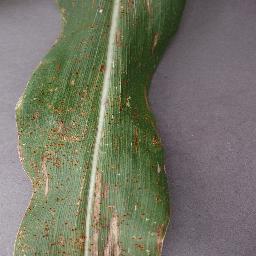

Supplement: S1 File — (ZIP) [file pone.0267650.s001.zip › All data sets/Anthracnose leaf blight/fcd349a6-5361-40b6-a650-f8319a6d1d58___RS_NLB3783.JPG]

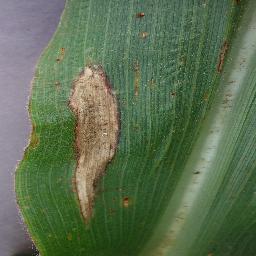

Supplement: S1 File — (ZIP) [file pone.0267650.s001.zip › All data sets/Anthracnose leaf blight/fcdc4514-7dfa-4003-aebd-27692e0853dd___RS_NLB3705.JPG]
